# Structurally Diverse Polycyclic Salicylaldehyde Derivative Enantiomers from a Marine-Derived Fungus *Eurotium* sp. SCSIO F452

**DOI:** 10.3390/md19100543

**Published:** 2021-09-26

**Authors:** Wei-Mao Zhong, Xiao-Yi Wei, Yu-Chan Chen, Qi Zeng, Jun-Feng Wang, Xue-Feng Shi, Xin-Peng Tian, Wei-Min Zhang, Fa-Zuo Wang, Si Zhang

**Affiliations:** 1 CAS Key Laboratory of Tropical Marine Bio-Resources and Ecology, Southern Marine Science and Engineering Guangdong Laboratory (Guangzhou), Guangdong Key Laboratory of Marine Materia Medica, RNAM Center for Marine Microbiology, South China Sea Institute of Oceanology, Chinese Academy of Sciences, 164 West Xingang Road, Guangzhou 510301, China; weimaozhong@hotmail.com (W.-M.Z.); 18489875310@163.com (Q.Z.); wangjunfeng@scsio.ac.cn (J.-F.W.); shixuefeng@scsio.ac.cn (X.-F.S.); xinpengtian@scsio.ac.cn (X.-P.T.); 2 Southwest Center for Natural Products Research, University of Arizona, Tucson, AZ 85706, USA; 3 University of Chinese Academy of Sciences, 19 Yuquan Road, Beijing 100049, China; 4 Key Laboratory of Plant Resources Conservation and Sustainable Utilization, South China Botanical Garden, Chinese Academy of Sciences, Guangzhou 510650, China; wxy@scbg.ac.cn; 5 State Key Laboratory of Applied Microbiology Southern China, Guangdong Provincial Key Laboratory of Microbial Culture Collection and Application, Guangdong Open Laboratory of Applied Microbiology, Institute of Microbiology, Guangdong Academy of Sciences, 100 Central Xianlie Road, Yuexiu District, Guangzhou 510070, China; chenyc@gdim.cn (Y.-C.C.); wmzhang@gdim.cn (W.-M.Z.)

**Keywords:** marine-derived fungi, *Eurotium* sp., natural products, salicylaldehyde derivative enantiomers, bioactivities

## Abstract

To enlarge the chemical diversity of *Eurotium* sp. SCSIO F452, a talented marine-derived fungus, we further investigated its chemical constituents from a large-scale fermentation with modified culture. Four pairs of new salicylaldehyde derivative enantiomers, euroticins F-I (**1**–**4**), as well as a known one eurotirumin (**5**) were isolated and characterized. Compound **1** features an unprecedented constructed 6/6/6/5 tetracyclic structures, while **2** and **3** represent two new types of 6/6/5 scaffolds. Their structures were established by comprehensive spectroscopic analyses, X-ray diffraction, ^13^C NMR, and electronic circular dichroism calculations. Selected compounds showed significant inhibitory activity against α-glucosidase and moderate cytotoxic activities against SF-268, MCF-7, HepG2, and A549 cell lines.

## 1. Introduction

Natural product discovery from marine-derived fungi has attracted more and more attention in recent decades. An increasing number of secondary metabolites with intriguing scaffolds and promising bioactivities have been isolated and characterized from marine fungi [1,2,3]. However, an inevitable problem of duplicated isolation of some common compounds has also emerged, resulting in wasted of time and labor. To improve the discovery efficiency of novel skeletal compounds, various strategies like genome mining and heterologous expression have been developed [4]. These strategies focus on manipulating genes responsible for the synthesis of secondary metabolites and employing heterologous expression or in vitro enzymatic studies to mine novel scaffolds. However, gene manipulation and bioinformatic analysis may deter natural-product chemists. Therefore, more traditional approaches, including One Strain Many Compounds (OSMAC) and large-scale culture are employed to enlarge the chemical space of microorganism [5]. The OSMAC approach emphasizes environmental alteration, like changing the culture medium, adding epigenetic regulators, and adjusting temperature and pH to trigger silent biosynthetic gene clusters expression and explore their chemical diversity, without the need to sequence and manipulate genes [5,6]. Another practical method is large-scale culture, which received increasing attention recently. This strategy focuses on the discovery of novel compounds in trace yield under normal scale incubation. These less explored minor compounds tend to be “dark matters” that bear novel skeletons. By scaling up, they can be better accumulated and isolable [5,7]. Therefore, it can serve as a complementary method to OSMAC, enabling natural product chemists to access more chemical entities.

**Figure 1:**
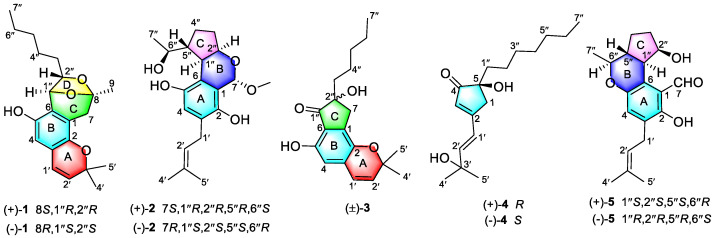
Structures of compounds **1**–**5**.

Our group has long been concentrated on unveiling bioactive secondary metabolites from marine-derived fungi [8,9,10,11]. Recently, we discovered different types of enantiomeric compounds, including spirocyclic diketopiperazine alkaloids, anthraquinones, and salicylaldehyde derivatives from *Eurotium* sp. SCSIO F452, a fungus isolated from a South China Sea sediment sample [12,13,14,15,16]. In order to enlarge the chemical space of this strain, we launched a 10 folds large-scale fermentation (300 L) with a modified liquid culture media and investigated its chemical constituents. A series of unprecedented salicylaldehyde derivative enantiomers with 6/6/6/5/7 and 6/6/6/6 polycyclic chemical scaffolds were isolated and characterized, which warrants an in-depth investigation of this sample [17,18]. Interestingly, we further discovered four pairs of new salicylaldehyde derivative enantiomers, namely euroticins F-I (**1**–**4**), with different polycyclic architectures, as well as a known one, eurotirumin [19] (**5**) (Figure 1), with its chiral separation and absolute configuration confirmed for the first time. Herein we report their isolation, structural elucidation, proposed biosynthetic pathway, and bioactivity evaluation.

## 2. Results

### 2.1. Structure Identification

Euroticin F (**1**) was isolated as a yellow solid. Its molecular formula C_21_H_28_O_4_ was assigned by HRMS (ESI) *m*/*z*: [M − H]^−^ Calcd for C_21_H_27_O_4_ 343.1915; Found 343.1922, corresponding to eight degrees of hydrogen deficiency. Its ^1^H NMR (Table 1) spectrum measured in DMSO-*d*_6_ exhibited four methyls at *δ*_H_ 0.86 (t, *J* = 6.7 Hz), 1.29 (s), 1.31 (s), 1.53 (s); five methylenes at *δ*_H_ 1.27 (overlap), 1.28 (overlap), 1.32 (overlap), 1.45 (m), 2.49 (overlap), 2.66 (d, *J* = 17.3 Hz); five methines at *δ*_H_ 3.75 (t, *J* = 6.7 Hz), 5.09 (s), 5.67 (d, *J* = 9.7 Hz), 6.27 (d, *J* = 9.7 Hz), 6.36 (s); one exchangeable proton at *δ*_H_ 8.99 (s). Its ^13^C NMR (Table 1) recorded 21 carbon resonances, including four methyls, five methylenes, five methines (two oxygenated, three olefinic), and seven nonprotonated carbons (five olefinic including two oxygenated, two aliphatic oxygenated). These NMR data partially resemble those of (±)-eurotiumide F [20], indicating **1** to be a salicylaldehyde analogue.

**Figure 2:**
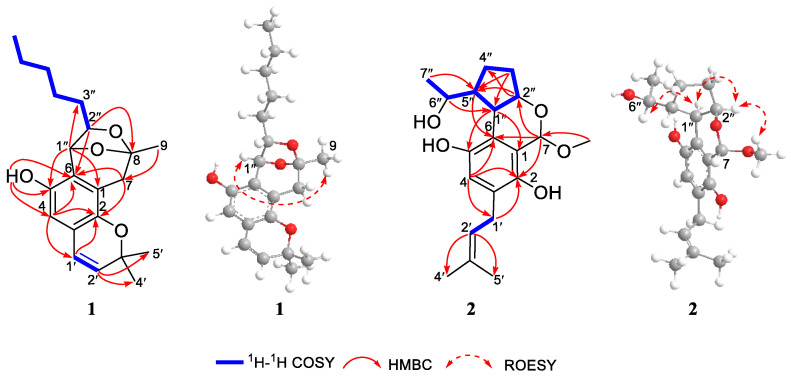
Key ^1^H-^1^H COSY, HMBC, and ROESY correlations of compounds **1** and **2**.

Rings A and B can be elucidated as a 2,2-dimethyl-2*H*-chromene based on HMBC and COSY correlations (Figure 2). A 3,6-dihydro-2*H*-pyran ring C was deduced to fuse with ring B via C-1 and C-6 by HMBC correlations from H_2_-7 (*δ*_H_ 2.49, overlap; 2.66, d, *J* = 17.3 Hz) to C-1 (*δ*_C_ 120.6), C-2 (*δ*_C_ 143.0), C-6 (*δ*_C_ 125.6), C-8 (*δ*_C_ 106.2); from H-1″ (*δ*_H_ 5.09, s) to C-1, C-5 (*δ*_C_ 145.7), C-6, C-8. Key HMBC correlations from H-2″ (*δ*_H_ 3.75, t, *J* = 6.7 Hz) to C-6, C-8, C-1″ (*δ*_C_ 74.1), as well as the chemical shift of C-8 indicated that C-8 and C-2″ (*δ*_C_ 84.8) are bridged by an oxygen atom Therefore, a 1,3-dioxolane ring D can be deduced to fuse ring C, forming a 7,8-dioxa-bicyclo[3.2.1]octene core, which is rare in salicylaldehyde compounds. A methyl group and an *n*-pentyl group are located at C-8 and C-2″, respectively, as elucidated by the HMBC correlations from H_3_-9 (*δ*_H_ 1.53, s) to C-7 (*δ*_C_ 35.8), C-8; from H-1″ to C-2″, C-3″ (*δ*_C_ 35.1); from H-2″to C-4″ (*δ*_C_ 25.2); as well as diagnostic ^1^H-^1^H COSY cross peaks of H-2″/H_2_-3″/H_2_-4″/H_2_-5″/H_2_-6″/H_3_-7″. Hitherto, the gross structure of **1** was established to be a polycyclic salicylaldehyde derivative with an unprecedented 6/6/6/5 skeleton.

**Figure 3:**
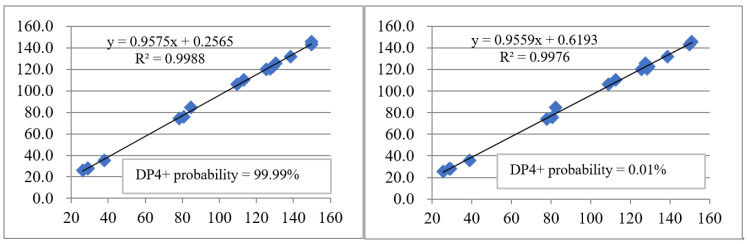
Linear regression analysis of calculated ^13^C NMR shifts of (8*R*,1″*S*,2″*S*)-**1** (**left**) and (8*R*,1″*S*,2″*R*)-**1** (**right**) against the measured shifts of **1** and the DP4+ probability for assignment of **1** to the candidate stereoisomers.

As for its relative configuration, the ROE correlation between H-1″ and H_3_-9 indicated their cofacial as *β*-orientations. Because there is no ^1^H-^1^H COSY correlation between H-1″ and H-2″, and H-1″ and H-2″ are displayed as sharp singlet and triplet in ^1^H NMR, respectively. The dihedral angle between H-1″ and H-2″ should tend to be 90 degree based on the Karplus equation, indicating that they are located on the opposite of the ring [21,22,23]. To further verify this conclusion, we calculated the ^13^C NMR spectra of (8*R*,1″*S*,2″*S*)-**1** and (8*R*,1″*S*,2″*R*)-**1** using gauge including atomic orbitals (GIAO) method at the mPW1PW91/6-311+G(d,p)/PCM(DMSO). The calculated ^13^C NMR data of (8*R*,1″*S*,2″*S*)-**1** (DP4+ probability: 99.99%) showed a better match with the measured data of **1** than that of (8*R*,1″*S*,2″*R*)-**1** (DP4+ probability: 0.01%) (Figure 3), which allowed the assignment of the relative configuration of 1 to be 8R*,1″S*,2″S*.

**Figure 4:**
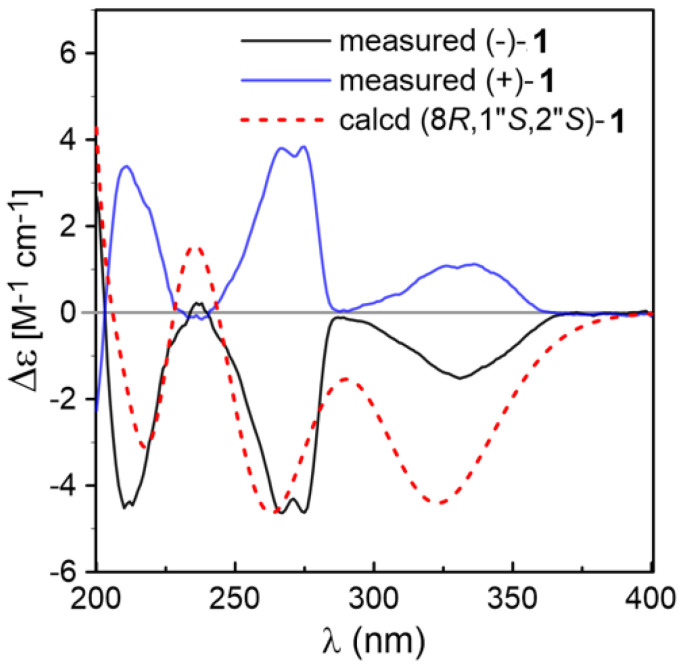
Comparison between M06/TZVP/PCM calculated and experimental ECD spectra of **1** in MeOH.

Due to the baseline circular dichroism and barely measurable optical rotation, compound **1** was deduced to be a racemate. It was separated by chiral HPLC to two optically pure enantiomers (+)-**1** and (−)-**1** (Figure S1). Subsequently, quantum chemical calculation of electronic circular dichroism (ECD) spectrum for (8*R*,1″*S*,2″*S*)-**1** matched well with that of measured for (−)-**1** (Figure 4). Finally, the absolute configuration of (+)-**1** and (−)-**1** could be determined as 8*S*,1″*R*,2″*R* and 8*R*,1″*S*,2″*S*, respectively.

**Figure 5:**
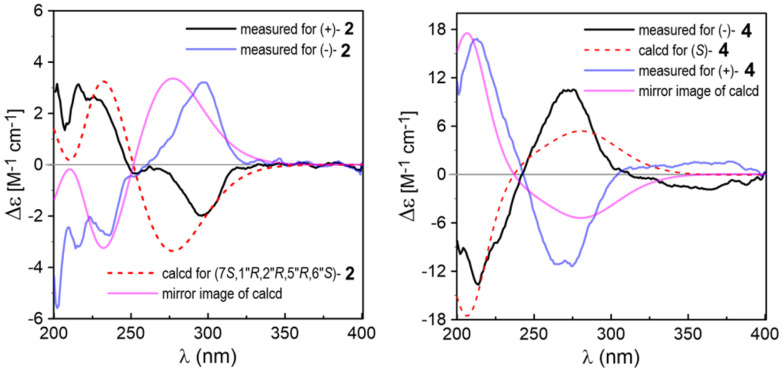
Comparison between PBE0/TZVP/PCM calculated and experimental ECD spectra of **2** and **4** in MeOH.

Euroticin G (**2**) was isolated as a yellow solid, with its molecular formula determined as C_20_H_28_O_5_ by HRMS (ESI) *m*/*z*: [M − H]^−^ Calcd for C_20_H_27_O_5_ 347.1864; Found 347.1871, indicating seven degrees of hydrogen deficiency. Its ^1^H NMR (Table 1) spectrum showed four methyls at *δ*_H_ 1.30 (d, *J* = 6.2 Hz), 1.69 (s), 1.72 (s), 3.45 (s), three methylenes at *δ*_H_ 1.39 (m), 1.71 (overlap), 1.92 (m), 2.49 (m), 3.27 (m), and seven methines at *δ*_H_ 1.33 (m), 2.30 (dd, *J* = 12.0, 8.1 Hz), 4.31 (dq, *J* = 10.4, 6.2 Hz), 4.61 (ddd, *J* = 8.1, 7.7, 5.0 Hz), 5.28 (br t, *J* = 7.4 Hz), 5.58 (s), 6.51 (s). The ^13^C NMR and DEPT (Table 1) revealed the presence of 20 carbon resonances, including four methyls (one oxygenated), three methylenes, seven methines (two olefinic, three oxygenated), six olefinic nonprotonated carbons (two oxygenated). Comparing these NMR data with eurotirumin (**5**) [19] shows that 2 has a different 6/6/5 tricyclic pattern. Detailed analysis of 2D NMR (Figure 2) reveals it has a prenylated aromatic A ring. A 3,4-dihydro-2*H*-pyran ring B was fused with ring A via C-1 and C-6 by HMBC correlations from H-7 (*δ*_H_ 5.58, s) to C-2 (*δ*_C_ 144.6), C-6 (*δ*_C_ 122.4), C-2″; from H-1″ (*δ*_H_ 2.30, dd, *J* = 12.0, 8.1 Hz) to C-1 (*δ*_C_ 121.1), C-5 (*δ*_C_ 148.2), C-6; as well as ^1^H-^1^H COSY cross peak of H-1″/H-2″. A methoxyl group is located at the acetal C-7 based on the HMBC correlation from OMe (*δ*_H_ 3.45, s) to C-7 (*δ*_C_ 95.9). Subsequently, a cyclopentane ring C was deduced to fuse with ring B by HMBC correlations from H_2_-3″ (*δ*_H_ 1.71, overlap; 2.49, m) to C-1″ (*δ*_C_ 41.3), C-5″ (*δ*_C_ 49.8); from H_2_-4″ (*δ*_H_ 1.39, m; 1.92, m) to C-1″, C-2″ (*δ*_C_ 67.9); from H-5″ (*δ*_H_ 1.33, m) to C-6, C-1″; as well as the diagnostic ^1^H-^1^H COSY cross peaks of H-2″/H_2_-3″/H_2_-4″/H-5″/H-1″. A CH_3_CHOH- side chain attached to C-5″ can be elucidated by HMBC correlations from H-6″ (*δ*_H_ 4.31, dq, *J* = 10.4, 6.2 Hz) to C-1″ and C-5″; from H_3_-7″ (*δ*_H_ 1.30, d, *J* = 6.2 Hz) to C-5″, C-6″ (*δ*_C_ 80.8); and ^1^H-^1^H COSY cross peaks of H-5″/H-6″/H_3_-7″. Careful analysis of the ROESY (Figure 2) of **2** revealed correlations between H-1″ and H-2″, H-2″ and 7-OMe, indicating their cofacial as *β*-orientations. Therefore, the coupling constant of *J*_H-1″/H-2″_ should be 8.1 Hz (Table 1). Then *J*_H-1″/H-5″_ should be assigned as 12.0 Hz, indicating H-1″/H-5″ to be *trans* relationship. From a biosynthetic point, the relative configuration of C-6″ in **2** was deduced to be *S* by sharing the same configuration with that in compound **5**, whose structure has been confirmed by X-ray experiment. Furthermore, conformation analysis revealed that there is a hydrogen bond between 6″-OH and 5-OH, which hinders the free rotation of C-5″/C-6″. When the relative configuration of C-6″ is assigned as *S*, its predominant configuration is coordinated with a diagnostic correlation between H-1″ and H-6″ observed in ROESY spectrum. But when C-6″ is *R*, it is not. Thus, the structure of **2** (Figure 1) was established with its relative configuration established as 7S*,1″R*,2″R*,5″R*,6″S*. This compound was proved to be a racemate by chiral HPLC separation (Figure S2), yielding (+)-**2** and (−)-**2**. The calculated ECD spectrum of 7*S*,1″*R*,2″*R*,5″*R*,6″*S*-**2** agreed well with that of measured for (+)-**2** (Figure 5). Therefore, the absolute configuration of (+)-**2** and (−)-**2** can be assigned as 7*S*,1″*R*,2″*R*,5″*R*,6″*“S* and 7*R*,1″*S*,2″*S*,5″*S*,6″*R*, respectively.

**Figure 6:**
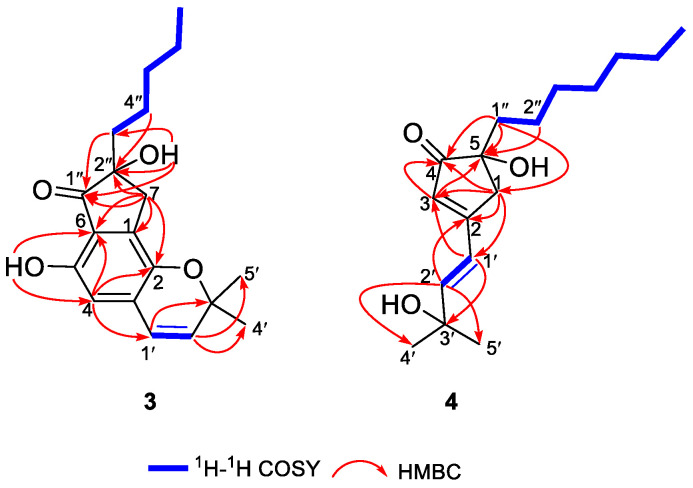
Key ^1^H-^1^H COSY and HMBC correlations of compounds **3** and **4**.

Euroticin H (**3**) was obtained as a yellow solid. Its molecular formula C_19_H_24_O_4_ was determined by HRMS (ESI) *m*/*z*: [M + H]^+^ Calcd for C_19_H_25_O_4_ 317.1747; Found 317.1748, indicating eight degrees of hydrogen deficiency. Its ^1^H NMR (Table 2) spectrum showed three methyls at *δ*_H_ 0.88 (t, *J* = 7.0 Hz), 1.42 (s), 1.43 (s); five methylenes at *δ*_H_ 1.29 (overlap), 1.30 (overlap), 1.35 (overlap), 1.53 (m), 1.83 (m), 2.11 (m), 4.81 (d, *J* = 16.3 Hz), 4.99 (d, *J* = 16.3 Hz); three methines at *δ*_H_ 6.07 (d, *J* = 9.9 Hz), 6.46 (d, *J* = 9.9 Hz), 6.60 (s); two exchangeable protons at *δ*_H_ 5.09 (br s) and 11.20 (s). The ^13^C NMR and DEPT (Table 2) revealed the presence of 19 carbon resonances, including three methyls, five methylenes, three olefic methines, and eight nonprotonated carbons (one carbonyl, five olefinic including two oxygenated, two aliphatic oxygenated). Comparing the NMR data of **3** with **1** indicated it has a similar partial structure of rings A and B. An *α*,*β*-unsaturated cyclopentanone was deduced to fuse with ring B via C-1 and C-6 by HMBC correlations (Figure 6) from H_2_-7 (*δ*_H_ 4.81, dd, *J* = 16.3 Hz; 4.99, dd, *J* = 16.3 Hz) to C-1 (*δ*_C_ 128.3), C-2 (*δ*_C_ 140.1), C-6 (*δ*_C_ 112.9), C-1″ (*δ*_C_ 197.5), C-2″ (*δ*_C_ 92.3). A hydroxyl group and an *n*-pentyl group are both located at C-2″, as elucidated by the HMBC correlations from 2″-OH (*δ*_H_ 5.90, br s) to C-1″, C-2″, C-3″ (*δ*_C_ 37.0); from H_2_-3″ (*δ*_H_ 1.83, m; 2.11, m) to C-1″, C-2″; from H_2_-4″ (*δ*_H_ 1.35, overlap; 1.53, m) to C-2″, C-3″; combined with diagnostic ^1^H-^1^H COSY cross peaks of H_2_-3″/H_2_-4″/H_2_-5″/H_2_-6″/H_3_-7″. Compound **3** was deduced to be a racemate due to its baseline ECD. We tried to separate it by chiral columns but failed. Therefore, it was established to be a racemic salicylaldehyde derivative with a new type of 6/6/5 skeleton.

Euroticin I (**4**) was obtained as a yellow oil. Its molecular formula C_17_H_28_O_3_ was determined by HRMS (ESI) *m*/*z*: [M + Na]^+^ Calcd for C_17_H_28_O_3_Na 303.1931; Found 303.1938, indicating four degrees of hydrogen deficiency. The ^1^H and ^13^C NMR data (Table 2) of **4** were partially similar with those of flavoglaucin [16]. The conservative aromatic nuclei in flavoglaucin was changed to an *α*,*β*-unsaturated cyclopentanone in **2**, which can be evidenced by the HMBC correlations (Figure 6) from H_2_-1 (*δ*_H_ 2.80, d, *J* = 17.8 Hz; 2.51, overlap) to C-2 (*δ*_C_ 169.4), C-3 (*δ*_C_ 126.7), C-4 (*δ*_C_ 209.5), C-5 (*δ*_C_ 76.4). A hydroxyl group located at C-5 can be elucidated by the chemical shift of C-5 (*δ*_C_ 76.4) and HMBC correlations from H_2_-1″ (*δ*_H_ 1.50, dt, *J* = 12.6, 3.9 Hz; 1.39, dt, *J* = 12.6, 4.8 Hz) to C-1 (*δ*_C_ 41.1), C-4, C-5. The prenyl group in flavoglaucin was transformed to a 3-hydroxyl-3-methylbutene group attached to C-2 in **4**, as elucidated by HMBC correlations from H-1′ (*δ*_H_ 6.68, d, *J* = 15.8 Hz) to C-1, C-2, C-3, C-2′ (*δ*_C_ 149.5), C-3′ (*δ*_C_ 69.5); from H-2′ (*δ*_H_ 6.49, d, *J* = 15.8 Hz) to C-2, C-3′, C-4′ (*δ*_C_ 29.5), C-5′ (*δ*_C_ 29.5). The double bond between C-1′ and C-2′ was assigned as *E* geometry by the large coupling constant (*J*_H-1′/H-2′_ = 15.8 Hz) [24]. It was confirmed to be a racemate and further separated to yield (+)-**4** and (−)-**4** (Figure S3). The calculated ECD of *S*-**4** fitted well that of (−)-**4** (Figure 5). Accordingly, (+)-**4** can be assigned as *R*. To be noted, this compound represents the first dearomatized prenylated salicylaldehyde derivative from fungus.

**Figure 7:**
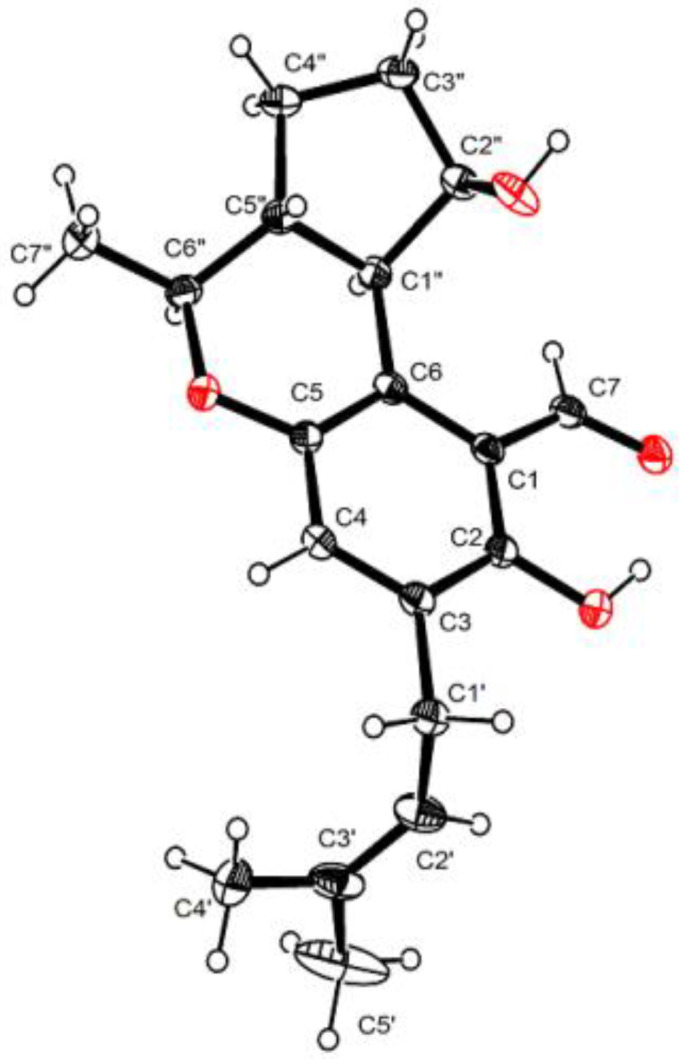
X-ray ORTEP drawing of compound (−)-**5**.

Compound **5** was isolated as a yellow solid. It was identified as eurotirumin [19] by comparison of HRMS and NMR data (Table S3). It was not demonstrated as a racemate or not. In our separation, we proposed it to be a racemate because of its low optical rotation. Then we successfully separated it by chiral HPLC (Figures S4 and S5) and fortunately obtained suitable crystals of (−)-**5** from MeOH for single-crystal X-ray diffraction experiment, which confirmed its planar and absolute configuration as 1″*R*,2″*R*,5″*R*,6″*S* with the Flack parameters of −0.06(15) (CCDC 2087463) (Figure 7). Thus, (−)-**5** can be assigned as 1″*S*,2″*S*,5″*S*,6″*R*.

### 2.2. Proposed Biosynthesis Pathway

**Scheme 1:**
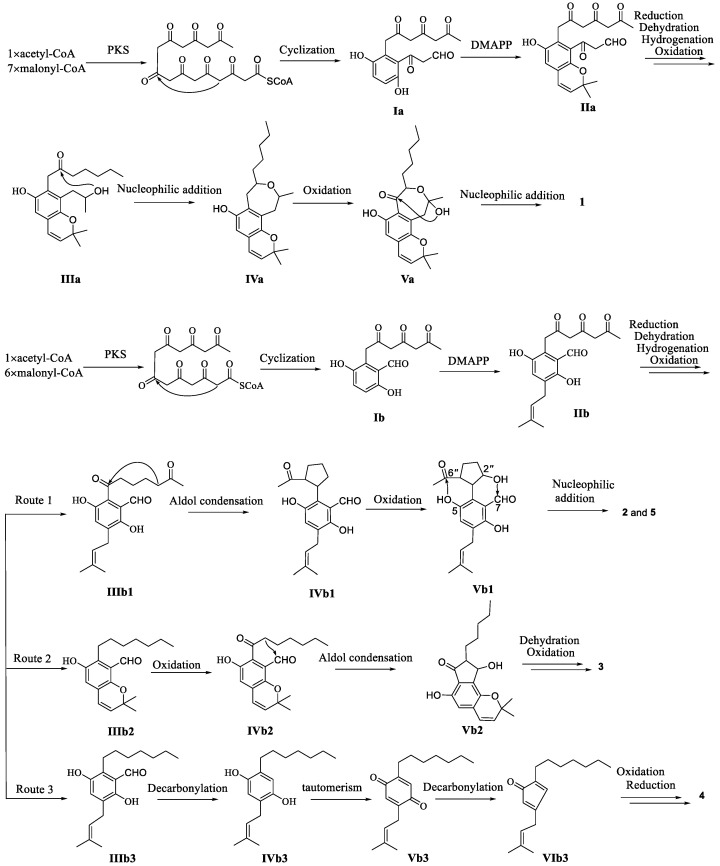
Proposed biosynthetic pathway of compounds **1**–**5**.

The putative biosynthetic pathway of **1**–**5** is proposed in Scheme 1. For **1**, intermediate **Ia** was formed by polyketide pathway from the starting precursor (one acetyl-CoA, seven malonyl-CoA) and cyclization. Then **Ia** underwent prenylation to produce **IIa**. A series of selective reduction, dehydration, hydrogenation, and oxidation of **IIa** could produce **IIIa**, which went through nucleophilic addition and oxidation to become **Va**. Another nucleophilic addition of **Va** would produce compound **1** [17] For **2**–**5**, a similar PKS pathway with one acetyl-CoA and six malonyl-CoA and prenylation gave **IIb**, which could generate three different precursors (**IIIb1**, **IIIb2**, **IIIb3**) for three different routes. In route 1, an aldol condensation of **IIIb1** furnished **IVb1**, which can be oxidized to **Vb1**. Subsequent nucleophilic addition occurred between 2″-OH and C-7 or 5-OH and C-6″ would produce compounds **2** and **5**, respectively. In route 2, **IIIb2** can transform to **Vb2** by oxidation and aldol concentration. Dehydration and oxidation of **Vb2** could yield compound **3**. In route 3, decarbonylation of **IIIb3** gave **IVb3** [25]. It can transform to **Vb3** by tautomerism with loss of one H_2_. Another decarbonylation of **Vb3** yielded **VIb3**, which went through oxidation and reduction to furnish compound **4**.

### 2.3. Bioactivity Evaluation

Compounds (+)-**1**, (−)-**1**, (+)-**2**, (−)-**2**, (±)**-3**, (+)-**4**, (−)-**4**, (+)-**5**, and (−)-**5** were evaluated for their in vitro cytotoxicity against four human cancer lines SF-268 (human glioblastoma carcinoma), MCF-7 (breast cancer), HepG-2 (liver cancer), and A549 (lung cancer), as well as antioxidative activity, *α*-glucosidase inhibitory, and antimicrobial activity against the bacteria *Staphylococcus aureus* and *Bacillus subtilis* [17,26]. All the compounds except (+)-**2**, (−)-**2**, and (±)-**3** exhibited moderate cytotoxic activities with IC_50_ values ranging from 12.74 to 55.5 μM (Table 3). Compounds (+)-**1**, (−)-**1**, (+)-**2**, (−)-**2** showed weak DPPH radical scavenging activity with EC_50_ values ranging from 41.40 to 77.07 μM (Table 4). Compounds (±)-**3** and (+)-**2** exhibited significant inhibitory against *α*-glucosidase with IC_50_ values of 16.31 ± 1.68 μM and 38.04 ± 2.73 μM, which are even better or comparative to that of the positive control Acarbose (IC_50_ of 32.92 ± 1.03 μM), while compounds (−)-**2** and (+)-**5** showed weak activity (IC_50_ of 89.41 ± 7.86 μM) (Table 4). None of these compounds exhibited antimicrobial activity at a concentration of 100 μM.

## 3. Materials and Methods

### 3.1. General Experimental Procedures

Optical rotations were measured with an MCP 500 automatic polarimeter (Anton Paar, Graz, Austria) with MeOH as solvent. UV spectra were recorded on a UV-2600 spectrometer (Shimadzu, Kyoto, Japan). Circular dichroism spectra were measured with a Chirascan circular dichroism spectrometer (Applied Photophysics, Ltd., Surrey, UK). Crystallographic data were collected on an XtaLAB AFC12 (Rigaku, Kyoto, Japan): Kappa single diffractometer using Cu K*α* radiation. ^1^H, ^13^C NMR, and 2D NMR spectra were recorded on the AVANCE 500 MHz and 700 MHz NMR with TMS as an internal standard the (Bruker Biospin GmbH, Rheinstetten, Germany). HRESIMS spectra data were recorded on a MaXis quadrupole-time-of-flight mass spectrometer (Bruker Biospin GmbH, Rheinstetten, Germany). Thin layer chromatography (TLC) was performed on plates precoated with silica gel GF254 (10–40 µm, Qingdao Marine Chemical Factory, Qingdao, China). Column chromatography (CC) was performed over silica gel (200–300 mesh and 300–400 mesh, Qingdao Marine Chemical Factory, Qingdao, China) and ODS (50 μM, YMC). High performance liquid chromatography was performed on an Agilent 1260 HPLC equipped with a DAD detector (Agilent, Technologies Co., Ltd., Palo Alto, CA, USA), using YMC-pack ODS-A column (250 × 10 mm, 5 µm, YMC CO., LTD., Kyoto, Japan) and COSMOSIL Cholester packed column (250 × 10 mm, 5 µm, Nacalai tesque, Kyoto, Japan). All solvents used in CC and HPLC were of analytical grade (Tianjin Damao Chemical Plant, Tianjin, China) and chromatographic grade (Oceanpak Alexative Chemical, Gothenburg, Sweden), respectively. Fractions were monitored by TLC and spots were visualized by heating silica gel plates sprayed with 10% H_2_SO_4_ in EtOH.

### 3.2. Fungal Material, Fermentation, and Extraction

As described previously [12].

### 3.3. Purification

The EtOAc extract (356 g) was subjected to a silica gel column using step gradient elution with CH_2_Cl_2_/Acetone (1:0 to 0:1) and yielded five fractions Frs.1-5 monitored by TLC. Fr.1 was separated by silica gel CC (PE/EtOAc 1:0 to 0:1) to obtain four subfractions (Frs.1.1–1.4). Fr.1.2 was separated by ODS CC with a gradient elution of MeOH/H_2_O (7:3 to 1:0) to produce six parts (Frs.1.2.1–1.2.6). Fr.1.2.1 was purified by silica gel CC (PE/EtOAc 1:0 to 0:1) to obtain four subfractions (Frs.1.2.1.1–1.2.1.4). Fr.1.2.1.1 was purified by HPLC (3 mL/min, 70% CH_3_CN/H_2_O, ODS-A column) to yield **1** (2.0 mg, *t*_R_ = 23.8 min). Fr.1.2.1.2 was purified by HPLC (3 mL/min, 69% CH_3_CN/H_2_O, ODS-A column) to yield **5** (4.5 mg, *t*_R_ = 18.2 min). Fr.1.2.1.3 was purified by HPLC (3 mL/min, 73% CH_3_CN/H_2_O, ODS-A column) to yield **2** (2.1 mg, *t*_R_ = 12.4 min). Fr.1.2.1.4 was purified by HPLC (3 mL/min, 67% CH_3_CN/H_2_O, Cholester column) to yield **3** (2.0 mg, *t*_R_ = 28.5 min). Fr.1.2.2 was purified by silica gel CC (PE/EtOAc 1:0 to 0:1) to obtain five subfractions (Frs.1.2.2.1–1.2.2.5). Fr.1.2.2.2 was purified by HPLC (3 mL/min, 43% CH_3_CN/H_2_O, Cholester column) to yield **4** (4.0 mg, *t*_R_ = 20.0 min).

Compounds **1**–**5** were isolated as racemates. Compound **1** was subjected to chiral HPLC (Daicel chiralpak IA, 250 × 4.6 mm, 5 μM) using n-hexane/isopropanol (*v*/*v*: 91:9; flow rate: 1 mL/min) as mobile phase to yield (+)-**1** (0.8 mg, *t*_R_ = 14.5 min) and (−)-**1** (0.8 mg, *t*_R_ = 9.1 min). Compound **2** was subjected to chiral HPLC (Daicel chiralpak IA, 250 × 4.6 mm, 5 μM) using *n*-hexane/isopropanol (*v*/*v*: 87:13; flow rate: 1 mL/min) to yield (+)-**2** (0.8 mg, *t*_R_ = 19.0 min) and (−)-**2** (0.8 mg, *t*_R_ = 12.7 min). Compound **4** was subjected to chiral HPLC (Daicel chiralpak IA, 250 × 4.6 mm, 5 μM) using *n*-hexane/isopropanol (*v*/*v*: 90:10; flow rate: 1 mL/min) to yield (+)-**4** (0.6 mg, *t*_R_ = 9.5 min) and (−)-**4** (0.6 mg, *t*_R_ = 11.2 min). Compound **5** was subjected to chiral HPLC (Daicel chiralpak IA, 250 × 4.6 mm, 5 μM) using *n*-hexane/isopropanol (*v*/*v*: 92:8; flow rate: 1 mL/min) to yield (+)-**5** (0.8 mg, *t*_R_ = 8.8 min) and (−)-**5** (0.8 mg, *t*_R_ = 10.3 min). Compound **3** was not separated successfully.

(±)-Euroticin F [(±)-**1**]. yellow solid; [*α*]25 D= 0 (*c* 0.1, MeOH); HRMS (ESI) *m*/*z*: [M − H]^−^ Calcd for C_21_H_27_O_4_ 343.1915; Found 343.1922. ^1^H and ^13^C NMR see Table 1.

(+)-**1**. yellow solid; [*α*]25 D= +91 (*c* 0.075, MeOH); ECD (MeOH) *λ*_max_ (Δ*ε*) 336 (+1.1), 275 (+3.8), 267 (+3.8), 211 (+3.4) nm.

(−)-**1**. yellow solid; [*α*]25 D= −90 (c 0.075, MeOH); ECD (MeOH) *λ*_max_ (Δ*ε*) 331 (−1.5), 275 (−4.6), 267 (−4.6), 213 (−4.5) nm.

(±)-Euroticin G [(±)-**2**]. yellow solid; [*α*]25 D= 0 (*c* 0.1, MeOH); UV (MeOH) *λ*_max_ (log *ε*) 296 (3.4), 206 (4.3) nm; HRMS (ESI) *m*/*z*: [M − H]^−^ Calcd for C_20_H_27_O_5_ 347.1864; Found 347.1871. ^1^H and ^13^C NMR see Table 1.

(+)-**2**. yellow solid; [*α*]25 D= +37 (*c* 0.1, MeOH); ECD (MeOH) *λ*_max_ (Δ*ε*) 296 (−2.0), 226 (+2.7), 216 (+3.1) nm.

(−)-**2**. yellow solid; [*α*]25 D= −35 (*c* 0.1, MeOH); ECD (MeOH) *λ*_max_ (Δ*ε*) 296 (+3.2), 236 (−2.8), 216 (−3.2) nm.

(±)-Euroticin H [(±)-**3**]. yellow solid; [*α*]25 D= 0 (*c* 0.02, MeOH); UV (MeOH) *λ*_max_ (log *ε*) 304 (3.8), 241 (3.8), 209 (3.8) nm; HRMS (ESI) *m*/*z*: [M + H]^+^ Calcd for C_19_H_25_O_4_ 317.1747; Found 317.1748. ^1^H and ^13^C NMR see Table 2.

(±)-Euroticin I [(±)-**4**]. yellow oil; [*α*]25 D= 0 (*c* 0.03, MeOH); UV (MeOH) *λ*_max_ (log *ε*) 273 (4.8) nm; HRMS (ESI) *m*/*z*: [M + Na]^+^ Calcd for C_17_H_28_O_3_Na 303.1931; Found 303.1938. ^1^H and ^13^C NMR see Table 2.

(+)-**4**. yellow oil; [*α*]25 D= +22 (*c* 0.06, MeOH); ECD (MeOH) *λ*_max_ (Δ*ε*) 362 (+1.4), 275 (−11.4), 213 (+16.9) nm.

(−)-**4**. yellow oil; [*α*]25 D= −22 (*c* 0.06, MeOH); ECD (MeOH) *λ*_max_ (Δ*ε*) 365 (−1.9), 276 (+10.5), 2714 (−13.7) nm.

(±)-eurotirumin [(±)-**5**]. yellow solid; [*α*]25 D= 0 (*c* 0.02, MeOH); UV (MeOH) *λ*_max_ (log *ε*) 274 (3.5), 202 (4.2) nm; HRMS (ESI) *m*/*z*: [M − H]^−^ Calcd for C_19_H_23_O_4_ 315.1602; Found 315.1607. ^1^H and ^13^C NMR see Table S1.

(+)-**5**. yellow solid; [*α*]25 D= +70.8 (*c* 0.05, MeOH); ECD (MeOH) *λ*_max_ (Δ*ε*) 318 (+0.9), 239 (−2.8) nm.

(−)-**5**. yellow solid; [*α*]25 D= −71.4 (*c* 0.05, MeOH); ECD (MeOH) *λ*_max_ (Δ*ε*) 312 (−1.1), 237 (+3.1) nm.

### 3.4. X-ray Crystallographic Analysis

Crystallographic data were collected on an XtaLAB AFC12 (Rigaku, Kyoto, Japan): Kappa single diffractometer using Cu K*α* radiation. The structure was solved with the ShelXT (Germany) structure solution program using Intrinsic Phasing and refined with the ShelXT refinement package using Least Squares minimization. The crystallographic data for (−)-**5** was deposited in the Cambridge Crystallographic Data Centre (CCDC deposition number: 2087463). The data can be obtained freely from the Cambridge Crystallographic Data Centre by visiting sites of www.ccdc.cam.ac.uk/conts/retrieving.html (accessed on 1 July 2021).

Crystal Data for Compound (−)-**5**: C_18.9875_H_23.9625_O_4_ (*M* = 316.19 g/mol): orthorhombic, space group P212121, *a* = 4.52320(10) Å, *b* = 10.1857(3) Å, *c* = 35.1507(9) Å, *V* = 1619.46(7) Å^3^, *Z* = 4, *T* = 100.0(10) K, *μ*(CuKα) = 0.726 mm^−1^, *D*_calc_ = 1.297 g/cm^3^, 6838 reflections measured (9.04° ≤ 2*θ* ≤ 148.452°), 3174 unique (*R*_int_ = 0.0331, *R*_sigma_ = 0.0418) which were used in all calculations. The final *R*_1_ was 0.0508 (*I* > 2*σ*(*I*)) and *wR*(*F*^2^) was 0.1386 (all data). The goodness of fit on *F*^2^ was 1.049. Flack parameter = −0.06(15).

### 3.5. ECD and ^13^C NMR Calculation Methods

The theoretical calculations of ^13^C NMR of **1** and ECD of **1**, **2**, and **4** were carried out using the Gaussian 09 [27] and ORCA 4.2.1 [28,29] software packages. Conformational analysis was initially performed using Spartan’14 (Wavefunction, Irvine, CA, USA). More details about the experimental procedures and the optimized conformation geometries, thermodynamic parameters, and populations of all conformations are provided in Supporting Information.

### 3.6. Cytotoxicity, Antioxidative, and α-Glucosidase Inhibitory Activity, and Antimicrobial Activity Assays

#### 3.6.1. Cytotoxicity Assay

The cells of SF-268 (human glioblastoma carcinoma), MCF-7 (breast cancer), HepG-2 (liver cancer), and A549 (lung cancer) were purchased from Stem Cell Bank, Chinese Academy of Sciences. The cells were cultured in DMEM medium (Gibco) containing 10% fetal bovine serum (Gibco) at 37 °C in a humidified atmosphere with 5% (*v*/*v*) CO_2_. The cells were incubated in cultural flasks until sub-confluent (~80%). Then, cells (180 μL) with a density of 3 × 10^4^ cells/mL of media were seeded onto 96-well plates and incubated for 24 h at 37 °C, 5% CO_2_. Subsequently, 20 μL of different concentrations of compounds ranging from 1 to 128 μM in DMSO were added to each plate well. Equal volume of DMSO was used as a negative control. The plates were further incubated for 72 h. After incubation, cell monolayers were fixed with 50% (*wt*/*v*) trichloroacetic acid (50 μL) and stained for 30 min with 0.4% (*wt*/*v*) SRB dissolved in 1% acetic acid. Unbound dye was removed by washing repeatedly with 1% acetic acid. The protein-bound dye was dissolved in 10 mM Tris base solution (200 µL) for OD determination at 570 nm using a microplate reader. Adriamycin was used as positive control possessing potent cytotoxic activity. All data were obtained in triplicate and presented as means ± SD. IC_50_ values were calculated with the Sigma Plot 10.0 software (Systat Software Inc., CA, USA) using a non-linear curve-fitting method [17].

#### 3.6.2. Antioxidative Assay

Sample stock solutions (10 mM) were diluted to final concentrations of 2, 4, 8, 16, 32, 64, and 128 μM in ethanol. Then, 100 μL 0.2 mmol/L DPPH ethanol solution was added to 100 μL sample solutions of different concentrations on 96-well plates, and allowed to react at room temperature. Ascorbic acid was used as a positive control possessing potent antioxidant activity. After 12 h, the absorbance values were measured at 517 nm and converted into the percentage antioxidant activity (AA) using the following formula: AA(%) = [1 − (A_sample_ − A_blank_)/A_control_] × 100%. All data were obtained in triplicate and are presented as means ± S.D. EC_50_ values were calculated with the SigmaPlot 10.0 software (Systat Software Inc., CA, USA) using a non-linear curve-fitting method [17].

#### 3.6.3. α-Glucosidase Inhibitory Activity Assay

Inhibitory α-glucosidase activities were determined spectrophotometrically in a 96-well microtiter plate based on *p*-nitrophenyl-*α*-*D*-glucopyranoside (PNPG) as a substrate. In brief, 20 μL 0.2 U/mL *α*-glucosidase enzyme solution, 50 μL 0.1 mol/L PBS (pH 6.8), and 10 μL of the test compounds in DMSO were mixed and preincubated at 37 °C prior to initiation of the reaction by adding the substrate. After 10 min of preincubation, 20 μL 5 mmol/L PNPG solution was added and then incubated together at 37 °C. After 15 min of incubation, 20 μL 0.2 mol/L Na_2_CO_3_ was added to the test tubes to stop the reaction. The absorbance values were measured at 405 nm and converted into percentage inhibitory activity using the following formula: AA(%) = [1 − A_sample_/(A_negative control_ − A_blank_)] × 100%. Acarbose was used as positive control. All data were obtained in triplicate and presented as means ± SD. IC_50_ values were calculated with the Sigma Plot 10.0 software using a non-linear curve-fitting method [30].

#### 3.6.4. Antimicrobial Activity Assay

All the compounds were tested for antibacterial activity against *Staphylococcus aureus* and *Bacillus subtilis* by the Mueller–Hinton broth microdilution method. Tested strains were cultured for 16 h on a rotary shaker at 37 °C. Cultures were diluted with sterilized medium to achieve an optical absorbance of 0.04‒0.06 at 600 nm, then further diluted 10-fold before adding into 96-well microtiter plates. Compounds were dissolved in acetone, serially diluted to 7 concentrations (1.56–100 μg/mL), and tested in the 96-well plate in triplicate. The minimum inhibitory concentration (MIC) that completely inhibited visible growth of the tested strains were recorded after 18 h cultivation from three independent experiments, with vancomycin as the positive control and acetone as a blank control [26].

## 4. Conclusions

In conclusion, euroticins F-I (**1**–**4**), four pairs of new salicylaldehyde derivative enantiomers, as well as a known one, eurotirumin (**5**), were isolated from a South China Sea fungus *Eurotium* sp. SCSIO F452. Compound **1** features an unprecedented constructed 6/6/6/5 tetracyclic structures, while **2** and **3** represent two new types of 6/6/5 scaffolds. Compound **4** is the first dearomatized prenylated salicylaldehyde derivative. Compounds **1**–**5** are all occurred as racemates. Their optical pure enantiomers, except for **3** were well separated with absolute configuration unambiguously resolved by single crystal X-ray diffraction and ECD calculation for the first time. Selected compounds showed significant inhibitory activity against *α*-glucosidase and moderate cytotoxic activities. Our work would further enlarge the chemical diversity and pharmacological prosperity of salicylaldehyde derivatives.

## Figures and Tables

**Table 1:** ^1^H and ^13^C NMR Data for **1** in DMSO-*d*_6_ and **2** in acetone-*d*_6_ (700, 175, TMS, δ in ppm, *J* in Hz).

	1		2	
No.	*δ*_C_^a^	*δ*_H_ (*J*, Hz) ^b^	*δ*_C_^a^	*δ*_H_ (*J*, Hz) ^b^
1	120.6		121.1	
2	143.0		144.6	
3	120.1		127.7	
4	110.3	6.36, s	117.0	6.51, s
5	145.7		148.2	
6	125.6		122.4	
7	35.8	2.66, d (17.3)	95.9	5.58, s
		2.49, overlap		
8	106.2			
9	25.8	1.53, s		
1′	122.6	6.27, d (9.7)	29.2	3.27, m
2′	131.6	5.67, d (9.7)	123.8	5.28, br t (7.4)
3′	75.7		132.6	
4′	28.0	1.31, s	25.9	1.72, s
5′	27.8	1.29, s	17.8	1.69, s
1″	74.1	5.09, s	41.3	2.30, dd (12.0, 8.1)
2″	84.8	3.75, t (6.7)	67.9	4.61, ddd (8.1, 7.7, 5.0)
3″	35.1	1.45, m	35.0	2.49, m
				1.71, overlap
4″	25.2	1.32, overlap	29.5	1.92 m
				1.39, m
5″	31.5	1.27, overlap	49.8	1.33, m
6″	22.5	1.28, overlap	80.8	4.31, dq (10.4, 6.2)
7″	14.4	0.86, t (6.7)	21.8	1.30, d (6.2)
5-OH		8.99, s		
7-OMe			55.4	3.45, s

^a^ Recorded at 175 MHz. ^b^ Recorded at 700 MHz.

**Table 2:** ^1^H and ^13^C NMR data for **3** in acetone-*d*_6_ and **4** in DMSO-*d*_6_ (700, 175, TMS, δ in ppm, *J* in Hz).

	3		4	
No.	*δ*_C_^a^	*δ*_H_ (*J*, Hz) ^b^	*δ*_C_^c^	*δ*_H_ (*J*, Hz) ^d^
1	128.3		41.1	2.80, d (17.8)
				2.51, overlap
2	140.1		169.4	
3	130.8		126.7	6.01, s
4	112.9	6.60, s	209.5	
5	158.1		76.4	
6	112.9			
7	57.3	4.99, d (16.3)		
		4.81, d (16.3)		
1′	122.4	6.46, d (9.9)	121.7	6.68, d (15.8)
2′	138.0	6.07, d (9.9)	149.5	6.49, d (15.8)
3′	77.1		69.5	
4′	28.0	1.43, s	29.5	1.24, s
5′	27.7	1.42, s	29.5	1.24, s
1″	197.5		37.4	1.50, dt (12.6, 3.9)
				1.39, dt (12.6, 4.8)
2″	92.3		23.2	1.25 overlap
				1.10 m
3″	37.0	2.11, m	29.5	1.21, overlap
		1.83, m		
4″	23.6	1.53, m	28.7	1.22, overlap
		1.35, overlap		
5″	32.8	1.30, overlap	31.2	1.20, overlap
6″	23.2	1.29, overlap	22.1	1.24, overlap
7″	14.3	0.88, t (7.0)	14.0	0.84, t (7.2)
5-OH		11.2, s		
2″-OH		5.90, br s		

^a^ Recorded at 125 MHz. ^b^ Recorded at 500 MHz. ^c^ Recorded at 175 MHz. ^d^ Recorded at 700 MHz.

**Table 3:** Cytotoxic activities of compounds (+)-**1**, (−)-**1**, (+)-**2**, (−)-**2**, (±)-**3**, (+)-**4**, (−)-**4**, (+)-**5**, and (−)-**5** against tumor cells ^a^.

Compounds	IC_50_ (μM)			
	SF-268	MCF-7	HepG2	A549
(+)-**1**	21.88 ± 0.96	27.17 ± 2.03	28.00 ± 1.68	33.78 ± 0.34
(−)-**1**	37.31 ± 2.46	28.00 ± 1.57	30.72 ± 3.55	33.43 ± 1.50
(+)-**2**	>100	>100	>100	>100
(−)-**2**	>100	>100	>100	>100
(±)-**3**	>100	>100	>100	>100
(+)-**4**	12.74 ± 0.46	20.51 ± 1.54	19.88 ± 5.09	16.90 ± 0.92
(−)-**4**	23.73 ± 3.88	23.56 ± 2.99	19.53 ± 2.70	22.15 ± 1.54
(+)-**5**	21.98 ± 0.88	55.59 ± 4.85	41.18 ± 2.63	47.34 ± 0.71
(−)-**5**	35.65 ± 1.79	50.67 ± 2.48	40.69 ± 2.76	42.61 ± 1.43
Adriamycin	1.19 ± 0.03	2.02 ± 0.04	1.99 ± 0.07	1.73 ± 0.04

^a^ The results were mean ± SD (SD = standard deviation). Positive control: Adriamycin.

**Table 4:** Antioxidative and *α*-glucosidase inhibitory activities of compounds (+)-**1**, (−)-**1**, (+)-**2**, (−)-**2**, (±)-**3**, (+)-**4**, (−)-**4**, (+)-**5**, and (−)-**5**.

Compounds	Antioxidative Activity EC_50_ (μM)	*α*-Glucosidase Inhibitory IC_50_ (μM)
(+)-**1**	42.34 ± 3.85	>100
(−)-**1**	41.40 ± 2.68	>100
(+)-**2**	76.90 ± 0.99	38.04 ± 2.73
(−)-**2**	77.07 ± 1.88	79.71 ± 1.74
(±)-**3**	>100	16.31 ± 1.68
(+)-**4**	>100	>100
(−)-**4**	>100	>100
(+)-**5**	>100	89.41 ± 7.86
(−)-**5**	>100	>100
Ascorbic acid ^a^	11.35 ± 0.56	
Acarbose ^b^		32.92 ± 1.03

^a^ Positive control for antioxidative activity; ^b^ Positive control for *α*-glucosidase inhibitory activity.

## Data Availability

Data are contained within the article and Supplementary Materials.

## Supplementary Materials

The following are available online at https://www.mdpi.com/article/10.3390/md19100543/s1, Figure S1: The chiral HPLC chromatogram of **1**; Figure S2: The chiral HPLC chromatogram of **2**; Figure S3: The chiral HPLC chromatogram of **4**; Figure S4: The chiral HPLC chromatogram of **5**; Figure S5: Comparison between experimental ECD spectra of (+)-**5** and (−)-**5**; Figure S6: Structures of compounds applied for theoretical calculations; Figure S7: The ^1^H NMR (700 MHz) spectrum of euroticin F (**1**) in DMSO-*d*_6_; Figure S8: The ^13^C NMR (175 MHz) spectrum of euroticin F (**1**) in DMSO- *d*_6_; Figure S9: The HSQC (700 MHz) spectrum of euroticin F (**1**) in DMSO-*d*_6_; Figure S10: The HMBC (700 MHz) spectrum of euroticin F (**1**) in DMSO-*d*_6_; Figure S11: The ^1^H-^1^H COSY (700 MHz) spectrum of euroticin F (**1**) in DMSO-*d*_6_; Figure S12: The ROESY (700 MHz) spectrum of euroticin F (**1**) in DMSO-*d*_6_; Figure S13: The HRESIMS spectrum of euroticin F (**1**); Figure S14: The ^1^H NMR (700 MHz) spectrum of euroticin G (**2**) in acetone-*d*_6_; Figure S15: The ^13^C NMR (175 MHz) spectrum of euroticin G (**2**) in acetone-*d*_6_; Figure S16: The HSQC (700 MHz) spectrum of euroticin G (**2**) in acetone-*d*_6_; Figure S17: The HMBC (700 MHz) spectrum of euroticin G (**2**) in acetone-*d*_6_; Figure S18: The ^1^H-^1^H COSY (700 MHz) spectrum of euroticin G (**2**) in acetone-*d*_6_; Figure S19: The ROESY (700 MHz) spectrum of euroticin D (**2**) in acetone-*d*_6_; Figure S20: The HRESIMS spectrum of euroticin G (**2**); Figure S21: The UV spectrum of euroticin G (2); Figure S22: The ^1^H NMR (500 MHz) spectrum of euroticin H (**3**) in acetone-*d*_6_; Figure S23: The ^13^C NMR (125 MHz) spectrum of euroticin H (**3**) in acetone-*d*_6_; Figure S24: The HSQC (500 MHz) spectrum of euroticin H (**3**) in acetone-*d*_6_; Figure S25: The HMBC (500 MHz) spectrum of euroticin H (**3**) in acetone-*d*_6_; Figure S26: The ^1^H-^1^H COSY (500 MHz) spectrum of euroticin H (**3**) in acetone-*d*_6_; Figure S27: The HRESIMS spectrum of euroticin H (**3**); Figure S28: The HRESIMS spectrum of euroticin H (**3**); Figure S29: The ^1^H NMR (700 MHz) spectrum of euroticin I (**4**) in DMSO-*d*_6_; Figure S30: The ^13^C NMR (175 MHz) spectrum of euroticin I (**4**) in DMSO-*d*_6_; Figure S31: The HSQC (700 MHz) spectrum of euroticin I (4) in DMSO-*d*_6_; Figure S32: The HMBC (700 MHz) spectrum of euroticin I (**4**) in DMSO-*d*_6_; Figure S33: The ^1^H-^1^H COSY (700 MHz) spectrum of euroticin I (**4**) in DMSO-*d*_6_; Figure S34: The HRESIMS spectrum of euroticin I (**4**); Figure S35: The UV spectrum of euroticin I (**4**); Figure S36: The ^1^H NMR (700 MHz) spectrum of eurotirumin (**5**) in acetone-*d*_6_; Figure S37: The ^13^C NMR (175 MHz) spectrum of eurotirumin (**5**) in acetone-*d*_6_; Figure S38: The HSQC (700 MHz) spectrum of eurotirumin (**5**) in acetone-*d*_6_; Figure S39: The HMBC (700 MHz) spectrum of eurotirumin (**5**) in acetone-*d*_6_; Figure S40: The ^1^H-^1^H COSY (700 MHz) spectrum of eurotirumin (**5**) in acetone-*d*_6_; Figure S41: The ROESY (700 MHz) spectrum of eurotirumin (**5**) in acetone-*d*_6_; Figure S42: The HRESIMS spectrum of eurotirumin (**5**); Figure S43: The UV spectrum of eurotirumin (**5**); Table S1: Calculated relative thermal energies (Δ*E*), relative free energies (Δ*G*)*^a^*, and equilibrium populations (P)*^b^* of low-energy conformers (8*R*,1″*S*,2″*S*)-**1**, (8*R*,1″*S*,2″*R*)-**1**, (7*S*,1″*R*, 2″*R*,5″*R*,6″*S*)-**2,** (7*S*,1″*R*, 2″*R*,5″*R*,6″*R*)-**2**, and (*S*)-**4** in MeOH solution; Table S2: Calculated ^13^C NMR data for (8*R*,1″*S*,2″*S*)-**1** and (8*R*,1″*S*,2″*R*)-**1** and their goodness of fit with the measured shifts of **1**; Table S3: ^1^H and ^13^C NMR Data for **5** in acetone-*d*_6_ (700, 175, TMS, *δ* in ppm, *J* in Hz).
