# Supplementary material for: Structurally Diverse Polycyclic Salicylaldehyde Derivative Enantiomers from a Marine-Derived Fungus Eurotium sp. SCSIO F452
Source: Mar Drugs. 2021 Sep 26;19(10):543. doi: 10.3390/md19100543 (PMC8538301; doi:10.3390/md19100543)

Supporting Information

Structurally Diverse Polycyclic Salicylaldehyde Derivative Enantiomers from a Marine-Derived Fungus *Eurotium* sp. SCSIO F452

Wei-Mao Zhong [1,2,5], Xiaoyi Wei [3], Yu-Chan Chen [4], Qi Zeng [1,5], Jun-Feng Wang [1], Xue-Feng Shi [1], Xin-Peng Tian [1], Wei-Min Zhang [4], Fa-Zuo Wang [1,*] and Si Zhang [1,*]

1 CAS Key Laboratory of Tropical Marine Bio-resources and Ecology, Southern Marine Science and Engineering Guangdong Laboratory (Guangzhou), Guangdong Key Laboratory of Marine Materia Medica, RNAM Center for Marine Microbiology, South China Sea Institute of Oceanology, Chinese Academy of Sciences, 164 West Xingang Road, Guangzhou 510301, China; weimaozhong@hotmail.com (W.-M.Z.); 18489875310@163.com (Q.Z.); wangjunfeng@scsio.ac.cn (J.-F.W.); shixuefeng@scsio.ac.cn (X.-F.S.); xinpengtian@scsio.ac.cn (X.-P.T.); wangfazuo@scsio.ac.cn (F.-Z.W.); zhsimd@scsio.ac.cn (S.Z.)

2 Southwest Center for Natural Products Research, University of Arizona, Tucson, Arizona 85706, United States

3 Key Laboratory of Plant Resources Conservation and Sustainable Utilization, South China Botanical Garden, Chinese Academy of Sciences, Guangzhou 510650, China; wxy@scbg.ac.cn (X.-Y.W.)

4 State Key Laboratory of Applied Microbiology Southern China, Guangdong Provincial Key Laboratory of Mi-crobial Culture Collection and Application, Guangdong Open Laboratory of Applied Microbiology, Institute of Microbiology, Guangdong Academy of Sciences, 100 Central Xianlie Road, Yuexiu District, Guangzhou 510070, China; 454423583@qq.com (Y.-C.C.); wmzhang58@qq.com (W.-M.Z.)

5 University of Chinese Academy of Sciences, 19 Yuquan Road, Beijing 100049, China

* Corresponding authors:

Prof. Fazuo Wang

Tel: +86-20-3406-3746

E-mail: wangfazuo@scsio.ac.cn

Prof. Si Zhang

Tel: +86-20-8902-3103

E-mail: zhsimd@scsio.ac.cn

Table of Contents

Figure S1 The chiral HPLC chromatogram of **1**. 4

Figure S2 The chiral HPLC chromatogram of **2**.......... 4

Figure S3 The chiral HPLC chromatogram of **4**.......... 4

Figure S4 The chiral HPLC chromatogram of **5**.......... 4

Figure S5 Comparison between experimental ECD spectra of (+)-**5** and (-)-**5**. 5

Computational Details 5

Table S3 ^{1}H and ^{13}C NMR Data for **5** in acetone-*d*$_6$ (700, 175, TMS, *δ* in ppm, *J* in Hz).

Figure S7 The ^{1}H NMR (700 MHz) spectrum of euroticin F (**1**) in DMSO-*d*$_6$. 11

Figure S8 The ^{13}C NMR (175 MHz) spectrum of euroticin F (**1**) in DMSO-*d*$_6$.......... 11

Figure S9 The HSQC (700 MHz) spectrum of euroticin F (**1**) in DMSO-*d*$_6$. 12

Figure S10 The HMBC (700 MHz) spectrum of euroticin F (**1**) in DMSO-*d*$_6$. 12

Figure S11 The ^{1}H-^{1}H COSY (700 MHz) spectrum of euroticin F (**1**) in DMSO-*d*$_6$...... 13

Figure S12 The ROESY (700 MHz) spectrum of euroticin F (**1**) in DMSO-*d*$_6$.......... 13

Figure S13 The HRESIMS spectrum of euroticin F (**1**).......... 14

Figure S14 The ^{1}H NMR (700 MHz) spectrum of euroticin G (**2**) in acetone-*d*$_6$. 15

Figure S15 The ^{13}C NMR (175 MHz) spectrum of euroticin G (**2**) in acetone-*d*$_6$.......... 15

Figure S16 The HSQC (700 MHz) spectrum of euroticin G (**2**) in acetone-*d*$_6$. 16

Figure S17 The HMBC (700 MHz) spectrum of euroticin G (**2**) in acetone-*d*$_6$. 16

Figure S18 The ^{1}H-^{1}H COSY (700 MHz) spectrum of euroticin G (**2**) in acetone-*d*$_6$.... 17

Figure S19 The ROESY (700 MHz) spectrum of euroticin D (**2**) in acetone-*d*$_6$. 17

Figure S20 The HRESIMS spectrum of euroticin G (**2**).......... 18

Figure S21 The UV spectrum of euroticin G (**2**).......... 18

Figure S22 The ^{1}H NMR (500 MHz) spectrum of euroticin H (**3**) in acetone-*d*$_6$. 19

Figure S23 The ^{13}C NMR (125 MHz) spectrum of euroticin H (**3**) in acetone-*d*$_6$. 19

Figure S24 The HSQC (500 MHz) spectrum of euroticin H (**3**) in acetone-*d*$_6$.......... 20

Figure S25 The HMBC (500 MHz) spectrum of euroticin H (**3**) in acetone-*d*$_6$.......... 20

Figure S26 The ^{1}H-^{1}H COSY (500 MHz) spectrum of euroticin H (**3**) in acetone-*d*$_6$. .. 21

Figure S27 The HRESIMS spectrum of euroticin H (**3**).......... 21

Figure S28 The HRESIMS spectrum of euroticin H (**3**). 22

Figure S29 The ^{1}H NMR (700 MHz) spectrum of euroticin I (**4**) in DMSO-*d*$_6$. 22

Figure S30 The ^{13}C NMR (175 MHz) spectrum of euroticin I (**4**) in DMSO-d_6............ 23
Figure S31 The HSQC (700 MHz) spectrum of euroticin I (**4**) in DMSO-d_6................ 23
Figure S32 The HMBC (700 MHz) spectrum of euroticin I (**4**) in DMSO-d_6............... 24
Figure S33 The ^1H-^1H COSY (700 MHz) spectrum of euroticin I (**4**) in DMSO-d_6....... 24
Figure S35 The UV spectrum of euroticin I (**4**). 25
Figure S36 The ^1H NMR (700 MHz) spectrum of eurotirumin (**5**) in acetone-d_6......... 26
Figure S37 The ^{13}C NMR (175 MHz) spectrum of eurotirumin (**5**) in acetone-d_6........ 26
Figure S38 The HSQC (700 MHz) spectrum of eurotirumin (**5**) in acetone-d_6. 27
Figure S39 The HMBC (700 MHz) spectrum of eurotirumin (**5**) in acetone-d_6. 27
Figure S40 The ^1H-^1H COSY (700 MHz) spectrum of eurotirumin (**5**) in acetone-d_6.. 28
Figure S41 The ROESY (700 MHz) spectrum of eurotirumin (**5**) in acetone-d_6........... 28
Figure S42 The HRESIMS spectrum of eurotirumin (**5**). 29
Figure S43 The UV spectrum of eurotirumin (**5**)... 29

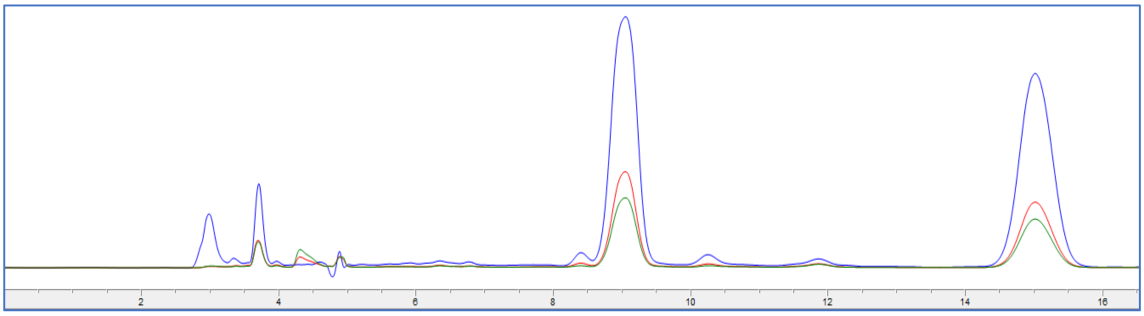

Figure S1 The chiral HPLC chromatogram of **1**.

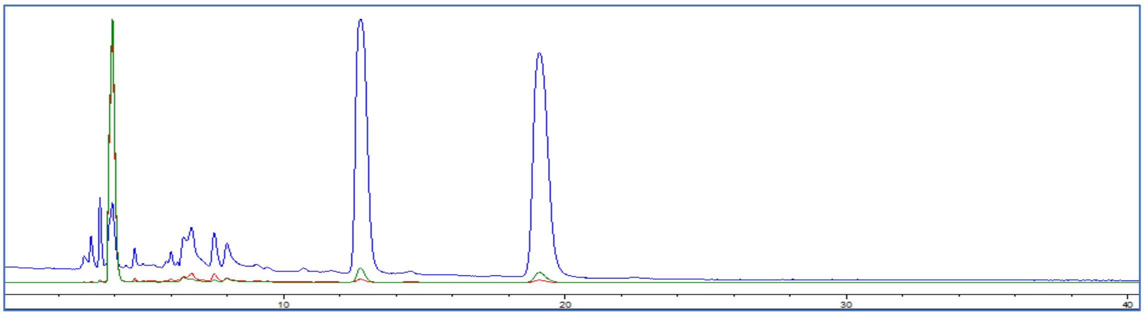

Figure S2 The chiral HPLC chromatogram of **2**.

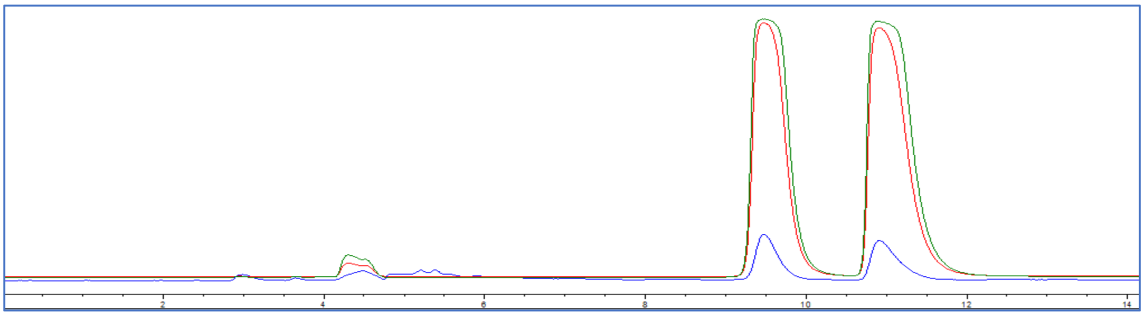

Figure S3 The chiral HPLC chromatogram of **4**.

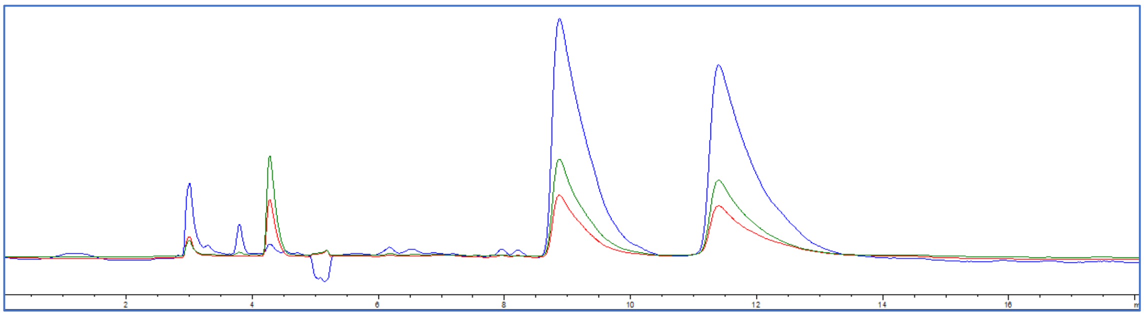

Figure S4 The chiral HPLC chromatogram of **5**.

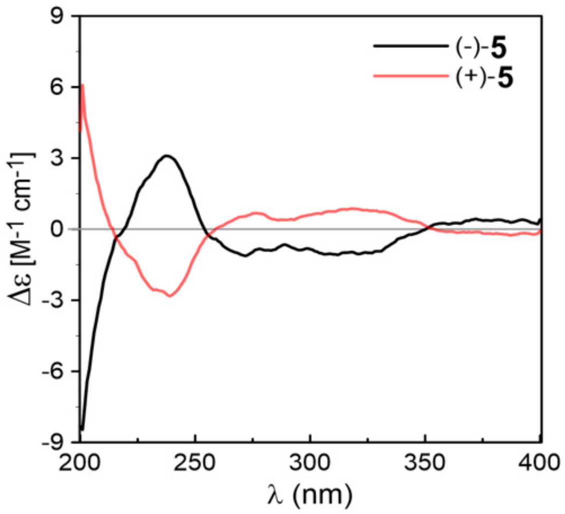

Figure S5 Comparison between experimental ECD spectra of (+)-**5** and (-)-**5**.

Computational Details

1. Methods

The Molecular Merck force field (MMFF) calculations were done using Spartan'14 program (Wavefunction Inc., Irvine, CA, USA). The density functional theory (DFT) and time-dependent density functional theory (TDDFT) calculations were performed with Gaussian09 program package.[1] Double-hybrid (DH) DFT calculations were conducted with ORCA 4.2.1 program package using RIJK approximation, tight SCF criteria, and grid 6 integrity.[2]

Truncated structures (8*R*,1″*S*,2″*S*)-**1**, (8*R*,1″*S*,2″*R*)-**1**, (7*S*,1″*R*,2″*R*,5″*R*,6″*S*)-**2**, (7*S*,1″*R*, 2″*R*,5″*R*,6″*R*)-**2**, and (*S*)-**4** were applied to theoretical computations. The MMFF conformers within 10 kcal/mol of the lowest energy conformer were subjected to DFT geometry optimizations at the B3LYP[3]/def2-SVP[4] level for **1**, and B3LYP-GD3BJ[5]/Def2-SVP level for **2** and **4**, respectively, both with the solvation model PCM for MeOH. Frequency calculations were run at the same corresponding levels to estimate their relative thermal (ΔE) and free energies (ΔG) at 298.15K. For **1**, energies of the low-energy conformers were re-calculated at the M06-2X[6]/def2-TZVP[4]/SMD(MeOH) level for **1**, and DH-DFT method at the PWPB95[7]-D3BJ/def2-TZVPP[4]/SMD(MeOH) level for **2** and **4**.

For calculations of the ^{13}C NMR shifts, low-energy conformers of the (8*R*,1″*S*,2″*S*)-**1** and (8*R*,1″*S*,2″*R*)-**1** were subjected to NMR calculations using the gauge including atomic orbitals (GIAO) method[8] at the mPW1PW91/6-311+G(d,p)/PCM(dmso) level.[9] The unscaled chemical shifts (δ_u) were computed using TMS as reference standard according to $\delta_u = \sigma_0 - \sigma^x$ (where σ^x is the Boltzmann averaged shielding tensor and σ_0 is the shielding tensor of TMS computed at the same level employed for σ^x). The Boltzmann averaging was done at 298.15 K using the relative energies obtained from the single-point NMR calculations. The goodness of fit between the predicted ^{13}C NMR data for

the two stereoisomers and the experimental data of compound **1** were evaluated by the modified DP4 probability (DP4+)[10], which was obtained using the unscaled shifts.

For simulations of the electronic circular dichroism (ECD) spectra, the TDDFT calculations were performed using the hybrid M06[6] and M06-2X functionals for (8*R*,1″*S*,2″*S*)-**1** and hybrid PBE1PBE (PBE0)[11] and M06-2X functionals for (7*S*,1″*R*,2″*R*,5″*R*,6″*S*)-**2** and (*S*)-**4**, both with Ahlrichs' basis set TZVP[12] and PCM solvent (MeOH) model. The calculated ECD spectra of individual conformers were generated by the program SpecDis[13] using a Gaussian band shape from dipole-length dipolar and rotational strengths. The equilibrium population of each conformer at 298.15K was calculated from its Δ*G* value using Boltzmann statistics. The overall ECD spectra were then generated according to the Boltzmann weighting of each conformer.

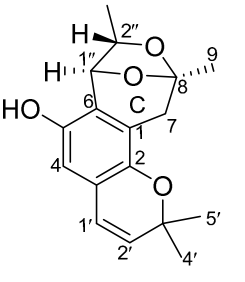

(8*R*,1″*S*,2″*S*)-**1**

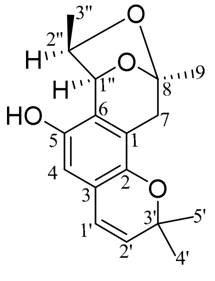

(8*R*,1″*S*,2″*R*)-**1**

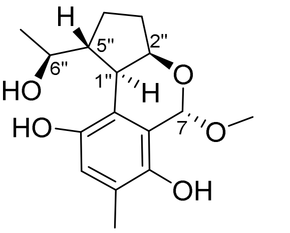

(7*S*,1″*R*,2″*R*,5″*R*,6″*S*)-**2**

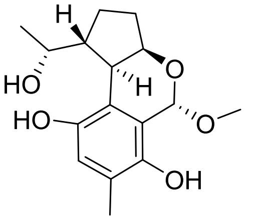

(7*S*,1″*R*,2″*R*,5″*R*,6″*R*)-**2**

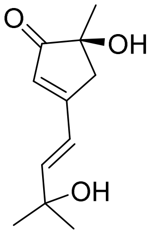

(*S*)-**4**

Figure S6. Structures of compounds applied for theoretical calculations.

2. Results

Table S1. Calculated relative thermal energies (Δ*E*), relative free energies (Δ*G*)[a], and equilibrium populations (P)[b] of low-energy conformers (8*R*,1″*S*,2″*S*)-**1**, (8*R*,1″*S*,2″*R*)-**1**, (7*S*,1″*R*,2″*R*,5″*R*,6″*S*)-**2,** (7*S*,1″*R*,2″*R*,5″*R*,6″*R*)-**2**, and (*S*)-**4** in MeOH solution.

conformer	Δ*E* (kcal/mol)	Δ*G* (kcal/mol)	P (%)
(8*R*,1″*S*,2″*S*)-**1**			
(8*R*,1″*S*,2″*S*)-**1a**	0.026	0.0	43.4
(8*R*,1″*S*,2″*S*)-**1b**	0.0	0.007	42.9
(8*R*,1″*S*,2″*S*)-**1c**	1.160	1.063	7.2
(8*R*,1″*S*,2″*S*)-**1d**	1.173	1.130	6.4
(8*R*,1″*S*,2″*R*)-**1**			
(8*R*,1″*S*,2″*R*)-**1a**	0.0	0.0	46.2

(8*R*,1″*S*,2″*R*)-**1b**	0.009	0.003	46.0
(8*R*,1″*S*,2″*R*)-**1c**	1.593	1.398	4.4
(8*R*,1″*S*,2″*R*)-**1d**	1.632	1.529	3.5
(7*S*,1″*R*, 2″*R*,5″*R*,6″*S*)-**2**			
(7*S*,1″*R*, 2″*R*,5″*R*,6″*S*)-**2a**	0.0	0.0	51.8
(7*S*,1″*R*, 2″*R*,5″*R*,6″*S*)-**2b**	0.601	0.418	25.6
(7*S*,1″*R*, 2″*R*,5″*R*,6″*S*)-**2c**	2.240	0.790	13.7
(7*S*,1″*R*, 2″*R*,5″*R*,6″*S*)-**2d**	1.10	1.073	8.5
(7*S*,1″*R*, 2″*R*,5″*R*,6″*R*)-**2**			
(7*S*,1″*R*, 2″*R*,5″*R*,6″*R*)-**2a**	0.688	0.0	31.9
(7*S*,1″*R*, 2″*R*,5″*R*,6″*R*)-**2b**	0.0	0.035	30.1
(7*S*,1″*R*, 2″*R*,5″*R*,6″*R*)-**2c**	1.308	0.516	13.4
(7*S*,1″*R*, 2″*R*,5″*R*,6″*R*)-**2d**	0.910	0.603	11.5
(7*S*,1″*R*, 2″*R*,5″*R*,6″*R*)-**2e**	0.762	0.828	7.9
(7*S*,1″*R*, 2″*R*,5″*R*,6″*R*)-**2f**	1.389	1.350	3.3
(7*S*,1″*R*, 2″*R*,5″*R*,6″*R*)-**2g**	1.625	1.647	2.0
(*S*)-**4**			
(*S*)-**4a**	0.0	0.0	65.7
(*S*)-**4b**	1.086	0.903	14.3
(*S*)-**4c**	1.075	0.845	15.8
(*S*)-**4d**	2.326	1.628	4.2

[a] Compound **1** at the M06-2X/def2-TZVP/SMD(MeOH)//B3LYP/def2-SVP/PCM(MeOH) level; compounds **2** and **4** at the PWPB95-D3/def2-tzvpp/SMD(MeOH)//B3LYP-GD3BJ/PCM(MeOH) level.

[b] From Δ*G* values at 298.15 K.

Table S2. Calculated ^{13}C NMR data for (8*R*,1″*S*,2″*S*)-**1** and (8*R*,1″*S*,2″*R*)-**1** and their goodness of fit with the measured shifts of **1**.

position	(8*R*,1″*S*,2″*S*)-**1**			(8*R*,1″*S*,2″*R*)-**1**			measured
	σ^x	δ_u	δ_s	σ^x	δ_u	δ_s	δ_{exp}
C-2	37.0	149.7	143.6	36.9	149.8	143.8	143.0
C-5	37.1	149.6	143.5	35.6	151.1	145.1	145.7
C-2′	47.9	138.8	133.1	48.0	138.7	133.2	131.6
C-6	56.0	130.7	125.4	58.9	127.8	122.7	125.6
C-1′	57.7	129.0	123.7	57.6	129.1	124.0	122.6
C-1	59.1	127.6	122.4	58.4	128.4	123.3	120.6
C-3	61.3	125.4	120.3	61.2	125.5	120.6	120.1
C-4	73.7	113.0	108.5	74.0	112.8	108.4	110.3
C-8	77.1	109.7	105.2	77.5	109.2	105.0	106.2
C-2″	102.0	84.7	81.3	104.3	82.4	79.4	84.8
C-3′	106.0	80.7	77.5	106.0	80.7	77.8	75.7
C-1″	108.5	78.2	75.1	108.9	77.8	75.0	74.1

C-7	148.7	38.0	36.6	147.7	39.1	38.0	35.8
C-4′	157.8	28.9	27.9	157.8	29.0	28.3	28.0
C-5′	157.8	28.9	27.9	157.8	28.9	28.2	27.8
C-9	160.5	26.2	25.3	161.3	25.4	24.9	25.8
Probability	sDP4+ = 99.63%<br>uDP4+ = 96.89%<br>DP4+ = 99.99%			sDP4+ = 0.37%<br>uDP4+ = 3.11%<br>DP4+ = 0.01%			

References

1 Frisch, M.J.; Trucks, G.W.; Schlegel, H.B.; Scuseria, G.E.; Robb, M.A.; Cheeseman, J.R.; Scalmani, G.; Barone, V.; Mennucci, B.; Petersson, G.A.; Nakatsuji, H.; Caricato, M.; Li, X.; Hratchian, H.P.; Izmaylov, A.F.; Bloino, J.; Zheng, G.; Sonnenberg, J.L.; Hada, M.; Ehara, M.; Toyota, K.; Fukuda, R.; Hasegawa, J.; Ishida, M.; Nakajima, T.; Honda, Y.; Kitao, O.; Nakai, H.; Vreven, T.; Montgomery, J.A.; Peralta, J.E.; Ogliaro, F.; Bearpark, M.; Heyd, J.J.; Brothers, E.; Kudin, K.N.; Staroverov, V.N.; Keith, T.; Kobayashi, R.; Normand, J.; Raghavachari, K.; Rendell, A.; Burant, J.C.; Iyengar, S.S.; Tomasi, J.; Cossi, M.; Rega, N.; Millam, J.M.; Klene, M.; Knox, J.E.; Cross, J.B.; Bakken, V.; Adamo, C.; Jaramillo, J.; Gomperts, R.; Stratmann, R.E.; Yazyev, O.; Austin, A.J.; Cammi, R.; Pomelli, C.; Ochterski, J.W.; Martin, R.L.; Morokuma, K.; Zakrzewski, V.G.; Voth, G.A.; Salvador, P.; Dannenberg, J.J.; Dapprich, S.; Daniels, A.D.; Farkas, O.; Foresman, J.B.; Ortiz, J.V.; Cioslowski, J.; Fox, D.J. Gaussian 09, revision D.01. Gaussian, Inc.: Wallingford CT, **2010**.

2 (a) Neese, F. The ORCA program system, *WIREs*: *Comput. Molecul. Sci.* **2012**, *2*, 73-78. (b) Neese, F. Software update: the ORCA program system, version 4.0, *WIREs*: *Comput. Molecul. Sci.* **2018**, *8*, e1327.

3 (a) Lee, C.; Yang, W.; Parr, R. G. Development of the Colle-Salvetti correlation-energy formula into a functional of the electron density. *Phys. Rev. B*. **1988**, *37*, 785-789; (b) Becke. A. D. Density-functional thermochemistry. III. The role of exact exchange. *J. Chem. Phys.* **1993**, *98*, 5648-5652.

4 Weigend, F.; Ahlrichs, R. Balanced basis sets of split valence, triple zeta valence and quadruple zeta valence quality for H to Rn: design and assessment of accuracy. *Phys. Chem. Chem. Phys.* **2005**, *7*, 3297-3305.

5 (a) Grimme, S.; Ehrlich, S.; Goerigk, L. Effect of the damping function in dispersion corrected density functional theory. *J. Comput. Chem.* **2011**, *32*, 1456-1465; (b) Grimme, S.; Antony, J.; Ehrlich, S.; Krieg, H. A consistent and accurate *ab initio* parametrization of density functional dispersion correction (DFT-D) for the 94 elements H-Pu. *J. Chem. Phys.* **2010**, *132*, 154104.

6 Zhao, Y.; Truhlar, D. G. The M06 suite of density functionals for main group thermochemistry, thermochemical kinetics, noncovalent interactions, excited states, and transition elements: two new functionals and systematic testing of four M06-class functionals and 12 other functionals. *Theor. Chem. Acc.* **2008**, *120*, 215-241.

7 Goerigk, L.; Grimme, S. Efficient and accurate double-hybrid-meta-GGA density functionals—evaluation with the extended GMTKN30 database for general main group thermochemistry, kinetics, and noncovalent Interactions. *J. Chem. Theory. Chem.* **2011**, *7*, 291-309.

8 (a) Wolinski, K.; Hinton, J. F.; Pulay, P. Efficient implementation of the gauge-independent atomic orbital method for NMR chemical shift calculations. *J. Am. Chem. Soc.* **1990**, *112*, 8251-8260; (b) Rohlfing, C. M.; Allen, L. C.; Ditchfield, R., Proton and carbon-13 chemical shifts: comparison between theory and experiment. *Chem. Phys* **1984**, *87*, 9-15; (c) Ditchfield, R. Self-consistent perturbation theory of diamagnetism: I. A gauge-invariant LCAO method for NMR chemical shifts. *Mol. Phys.* **1974**, *27*, 789-807; (d) Ditchfield, R. Molecular orbital theory of magnetic shielding and magnetic susceptibility. *J. Chem. Phys.* **1972**, *56*, 5688-5691.

9 Lodewyk, M. W.; Soldi, C.; Jones, P. B.; Olmstead, M. M.; Rita, J.; Shaw, J. T.; Tantillo, D. J. The correct structure of aquatolide experimental validation of a theoretically-predicted structural revision. *J. Am. Chem. Soc.* **2012**, *134*, 18550-18553.

10 (a) Grimblat, N.; Zanardi, M. M.; Sarotti, A. M. Beyond DP4: an improved probability for the stereochemical assignment of isomeric compounds using quantum chemical calculations of NMR shifts. *J. Org. Chem.* **2015**, *80*, 12526-12534; (b) Smith, S. G.; Goodman, J. M., Assigning stereochemistry to single diastereoisomers by GIAO NMR calculation: The DP4 probability. *J. Am. Chem. Soc.* **2010**, *132*, 12946-12959.

11 Adamo, C.; Barone, V. Toward reliable density functional methods without adjustable parameters: The PBE0 model. *J. Chem. Phys.* **1999**, *110*, 6158-6170.

12 Schäfer, A.; Huber, C.; Ahlrichs, R. Fully optimized contracted Gaussian basis sets of triple zeta valence quality for atoms Li to Kr. *J. Chem. Phys.* **1994**, *100*, 5829-5835.

13 Bruhn, T.; Schaumlöffel, A.; Hemberger, Y.; Bringmann, G. SpecDis: Quantifying the comparison of calculated and experimental electronic circular dichroism spectra. *Chirality* **2013**, *25*, 243-249.

Table S3 ^1H and ^{13}C NMR Data for **5** in acetone-d_6 (700, 175, TMS, δ in ppm, J in Hz).

	**5**	
No.	δ_C[a]	δ_H (J, Hz)[b]
1	118.7	
2	155.7	
3	130.1	
4	126.8	6.88, s
5	149.4	
6	123.3	
7	198.5	10.38, s
1′	27.7	3.27, m (7.0)
2′	122.5	5.31, br t (7.0)
3′	133.6	
4′	25.9	1.73, s
5′	17.8	1.70, s
1″	48.4	2.83, overlap
2″	73.9	4.78, br s
3″	36.0	2.40, m
		1.82, m
4″	24.8	1.89, m
		1.28, m
5″	32.5	2.14, m
6″	77.6	4.09, dq (10.2, 6.2)
7″	20.7	1.35, d (6.2)
2-OH		12.15, s

[a]Recorded at 175 MHz. [b]Recorded at 700 MHz.

Figure S7 The ^{1}H NMR (700 MHz) spectrum of euroticin F (**1**) in DMSO-d_6.

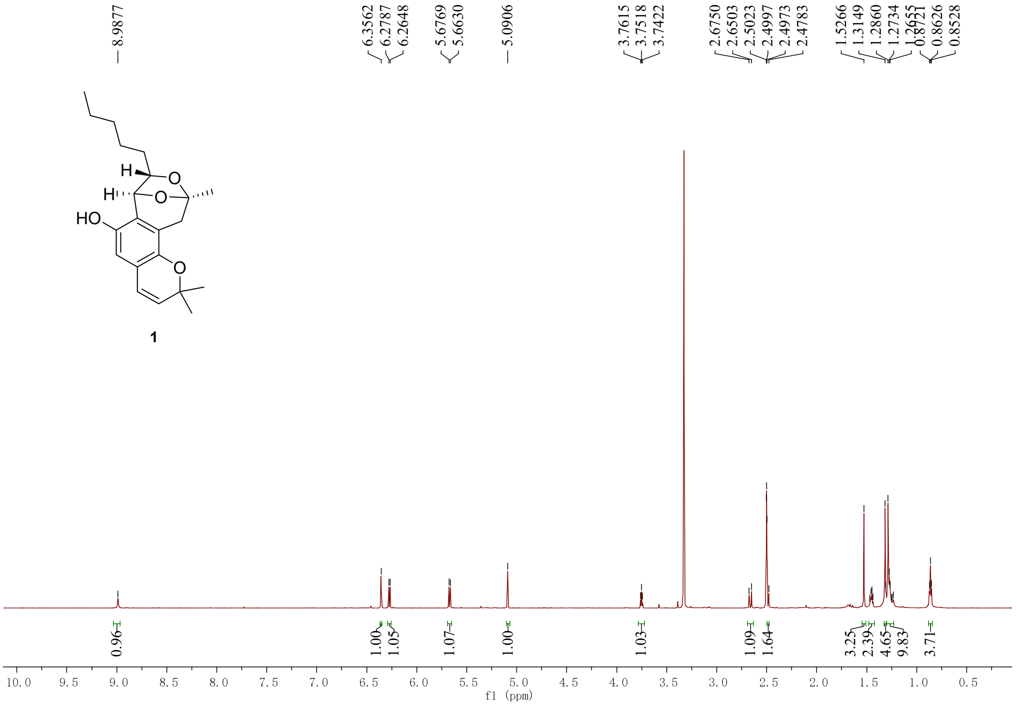

Figure S8 The ^{13}C NMR (175 MHz) spectrum of euroticin F (**1**) in DMSO-d_6.

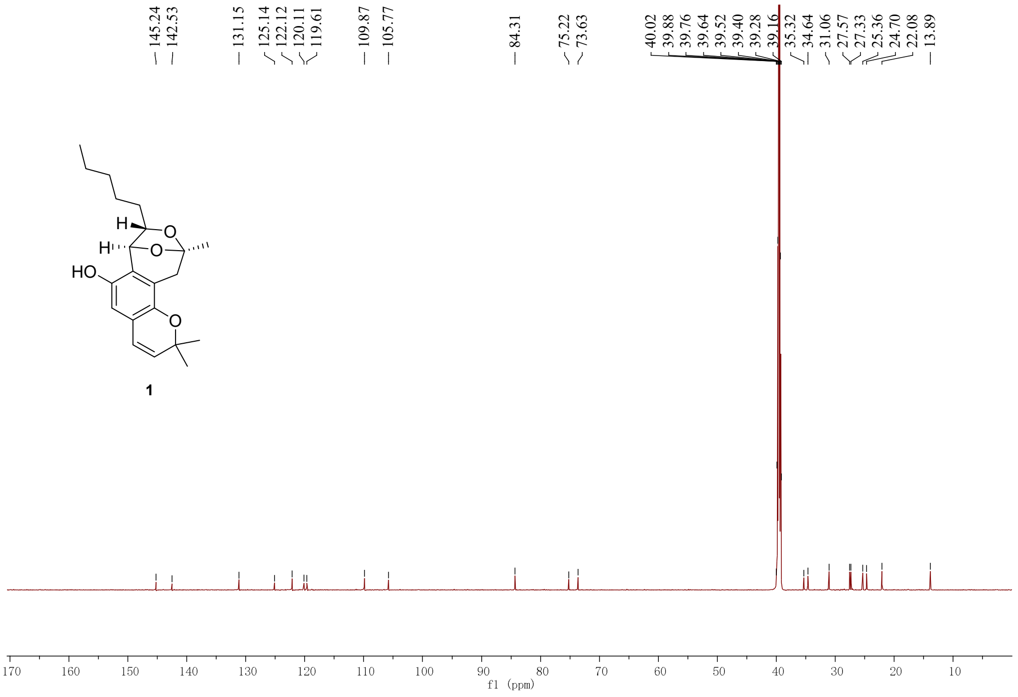

Figure S9 The HSQC (700 MHz) spectrum of euroticin F (**1**) in DMSO-*d*6.

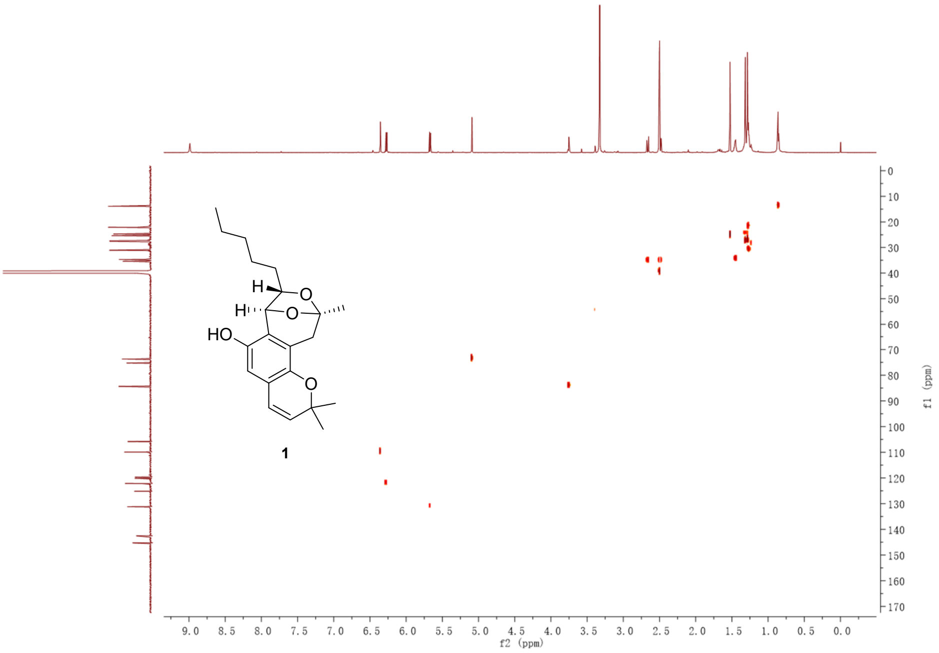

Figure S10 The HMBC (700 MHz) spectrum of euroticin F (**1**) in DMSO-*d*6.

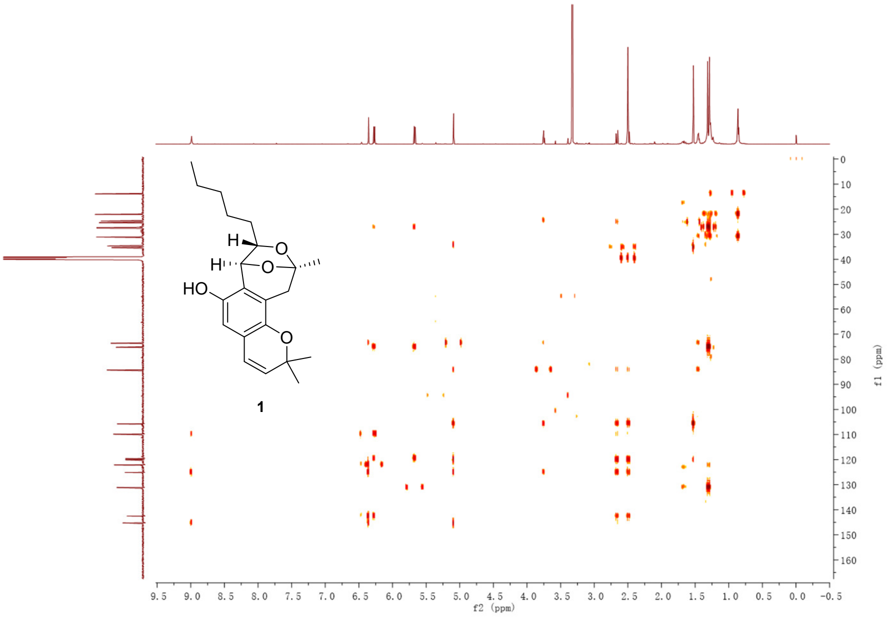

Figure S11 The ^{1}H-^{1}H COSY (700 MHz) spectrum of euroticin F (**1**) in DMSO-*d*6.

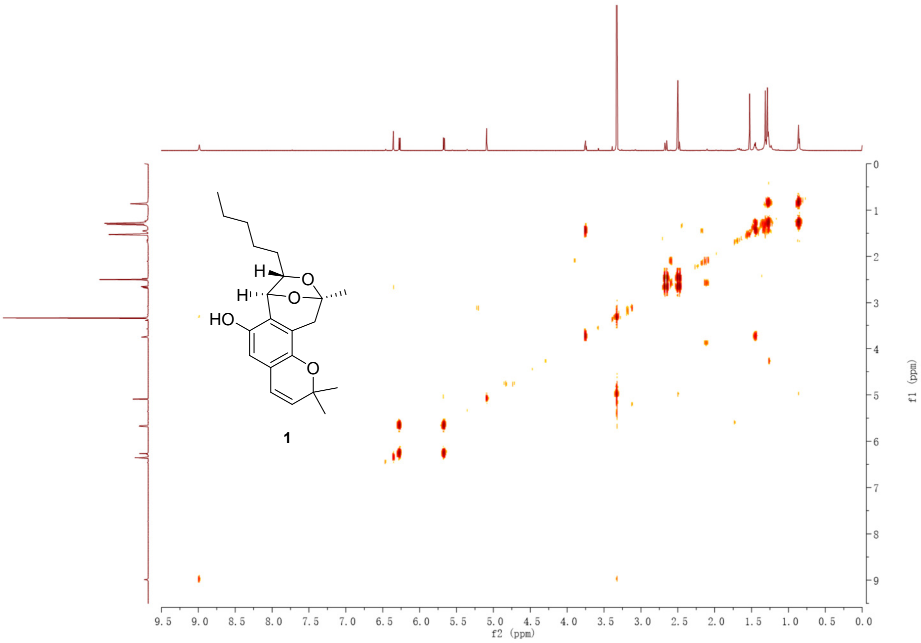

Figure S12 The ROESY (700 MHz) spectrum of euroticin F (**1**) in DMSO-*d*6.

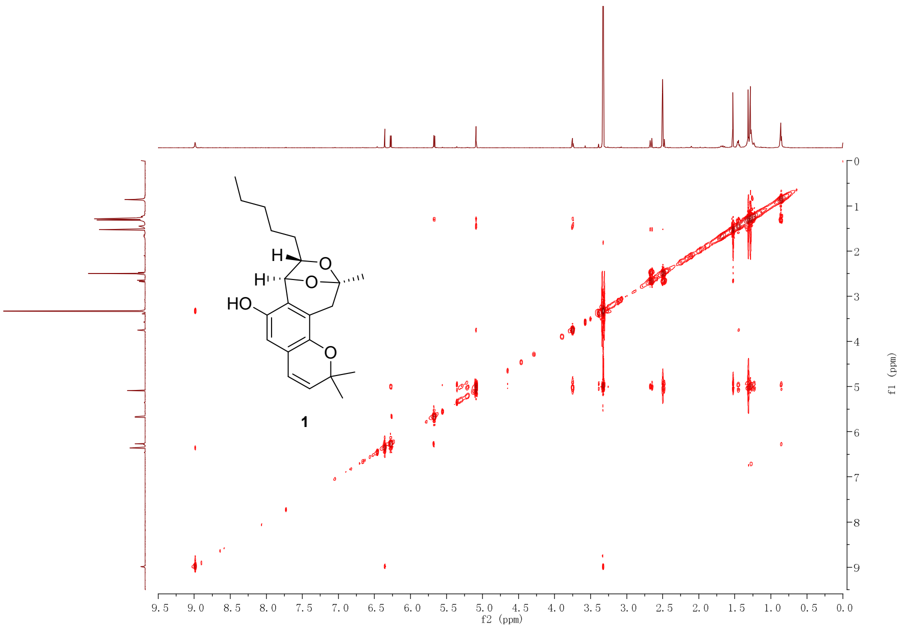

Figure S13 The HRESIMS spectrum of euroticin F (**1**).

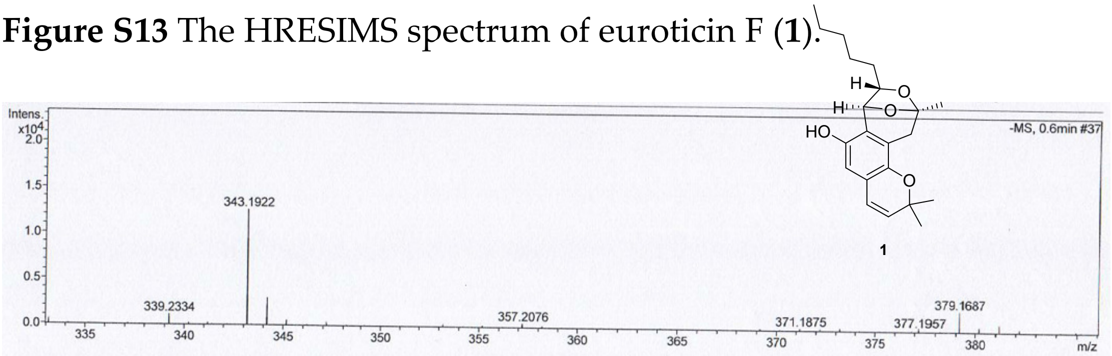

Meas. m/z	#	Ion Formula	Score	m/z	err [ppm]	err [mDa]	mSigma	rdb	e⁻ Conf	N-Rule
343.1922	1	C21H27O4	100.00	343.1915	-1.9	-0.7	6.2	8.5	even	ok
379.1687	1	C21H28ClO4	100.00	379.1682	1.5	0.6	12.2	7.5	even	ok
687.3915	1	C42H55O8	100.00	687.3902	1.9	1.3	26.1	15.5	even	ok
723.3700	1	C42H56ClO8	100.00	723.3669	-4.3	-3.1	39.3	14.5	even	ok

Figure S14 The ^{1}H NMR (700 MHz) spectrum of euroticin G (**2**) in acetone-d_6.

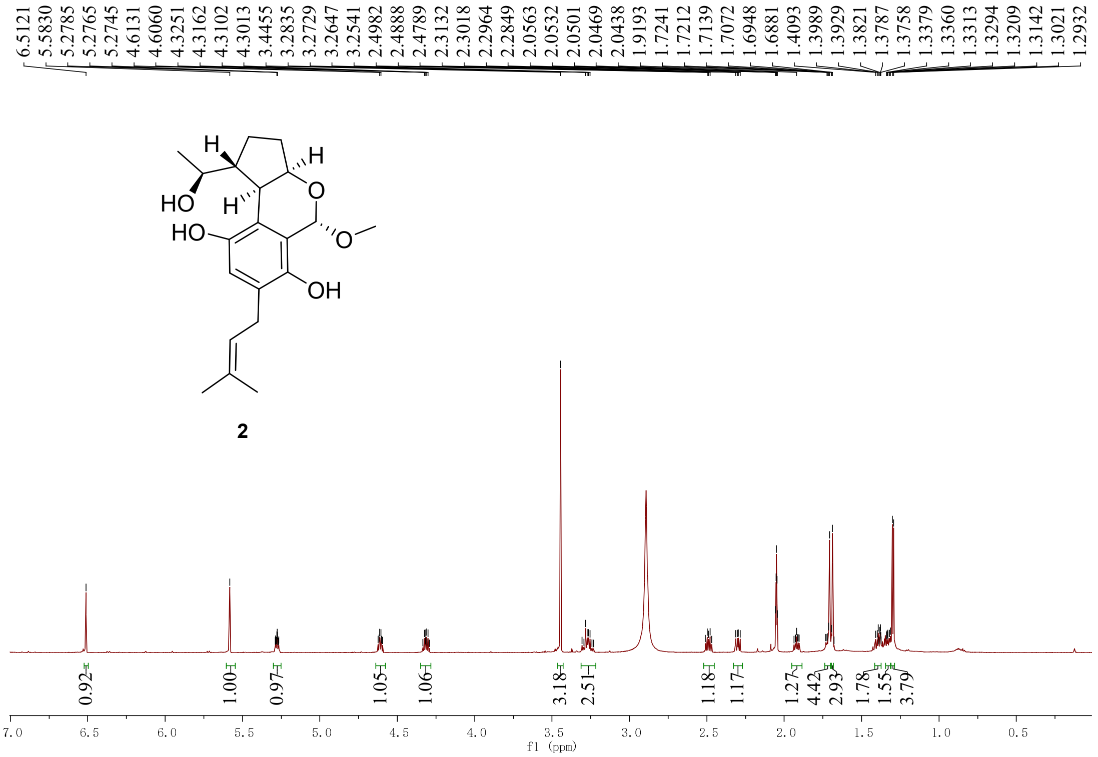

Figure S15 The ^{13}C NMR (175 MHz) spectrum of euroticin G (**2**) in acetone-d_6.

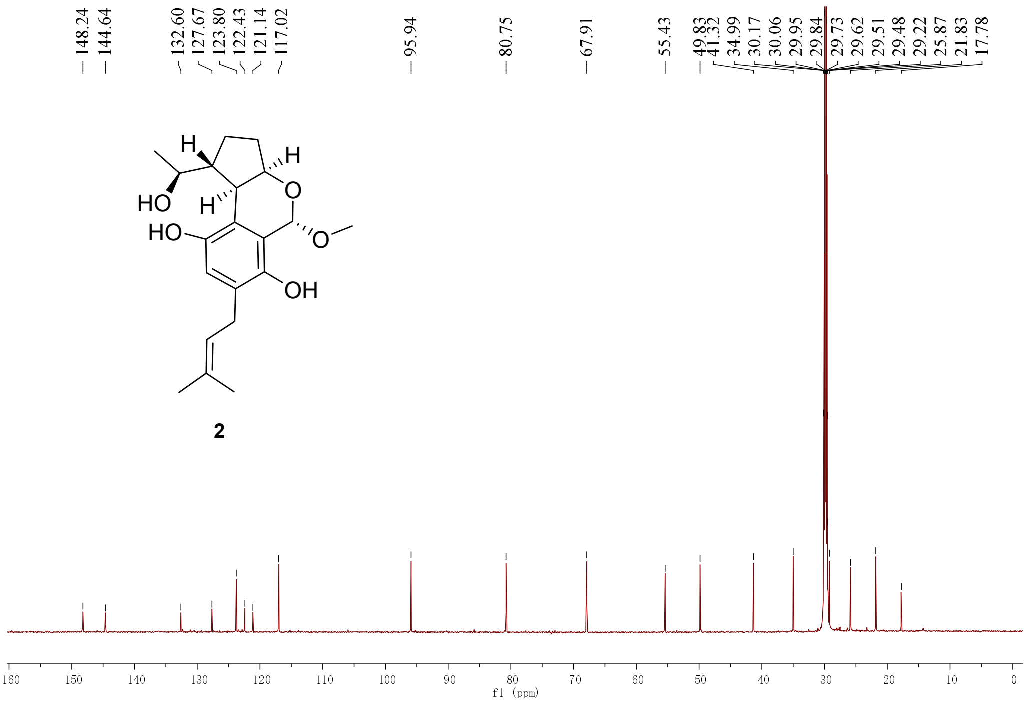

Figure S16 The HSQC (700 MHz) spectrum of euroticin G (**2**) in acetone-*d*6.

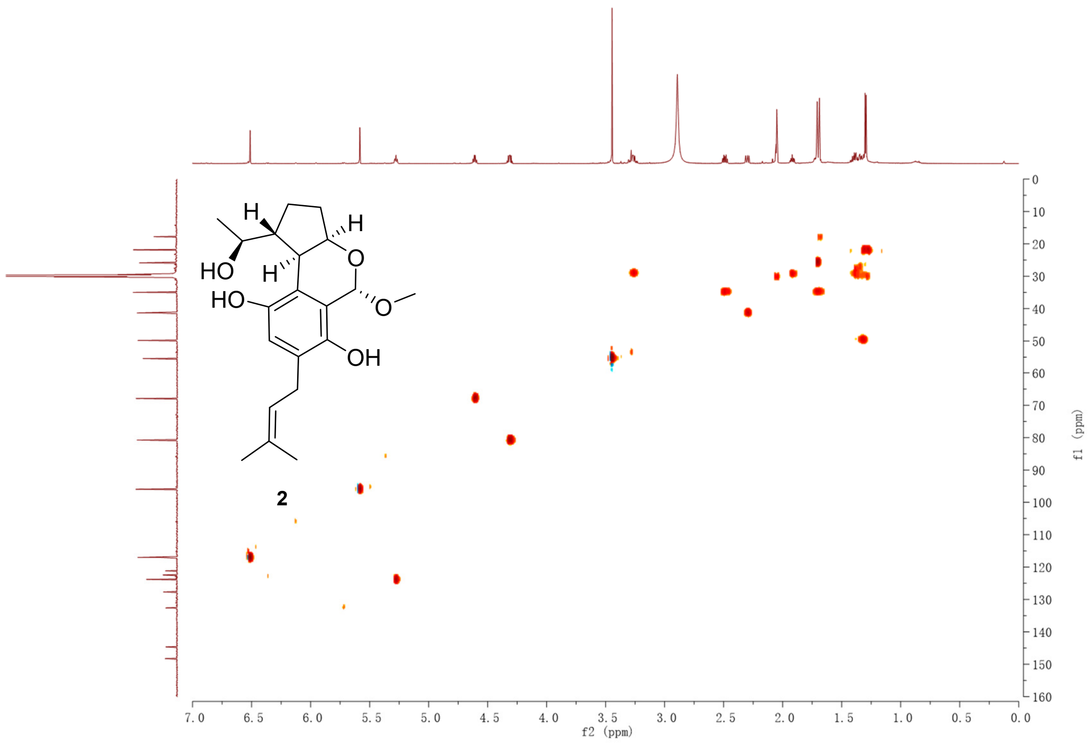

Figure S17 The HMBC (700 MHz) spectrum of euroticin G (**2**) in acetone-*d*6.

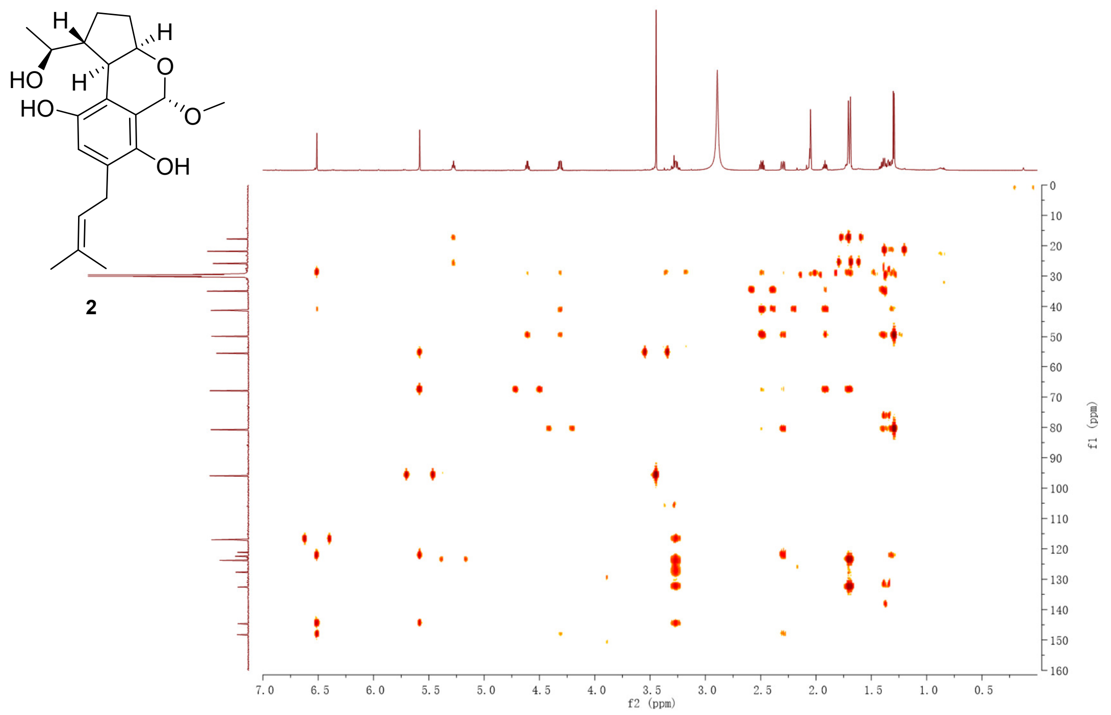

Figure S18 The ^{1}H-^{1}H COSY (700 MHz) spectrum of euroticin G (**2**) in acetone-d_6.

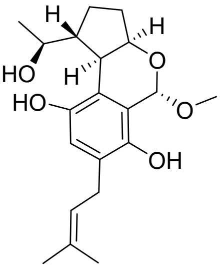

2

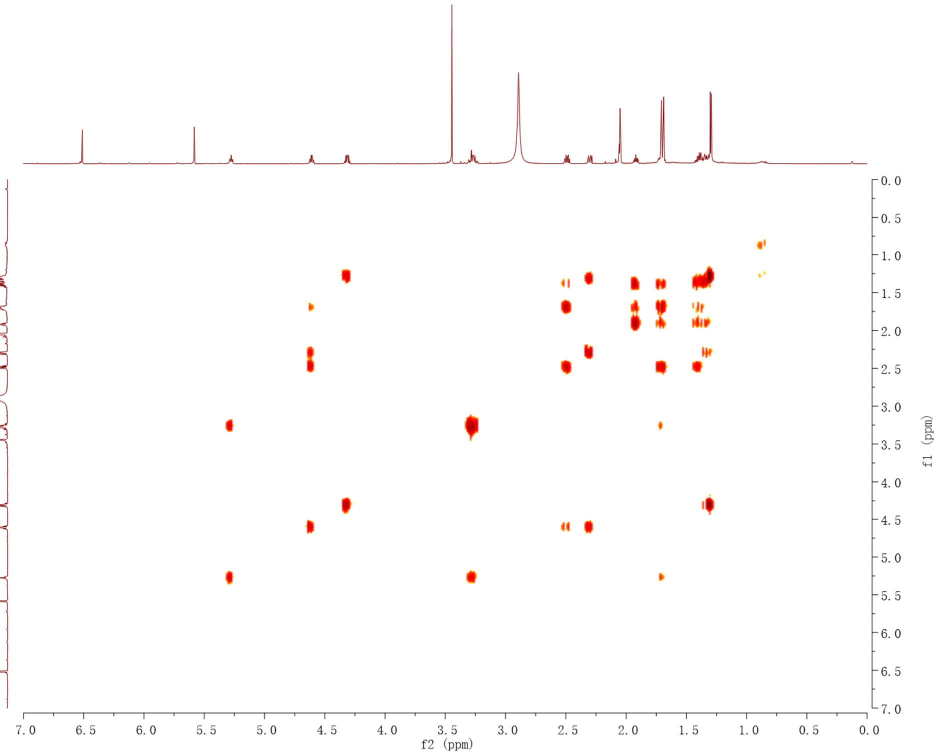

Figure S19 The ROESY (700 MHz) spectrum of euroticin D (**2**) in acetone-d_6.

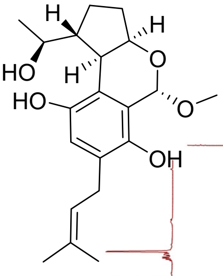

2

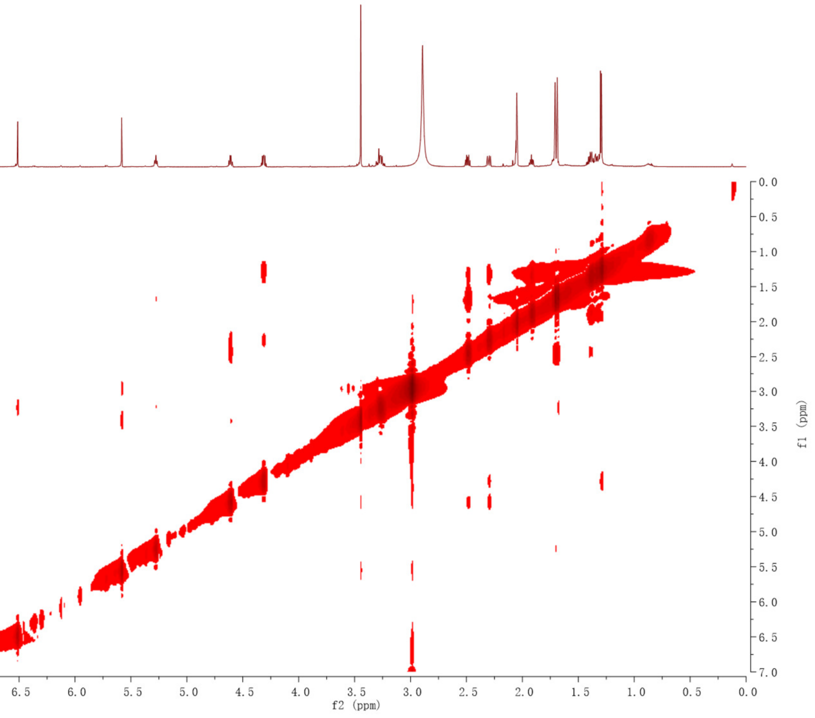

Figure S20 The HRESIMS spectrum of euroticin G (**2**).

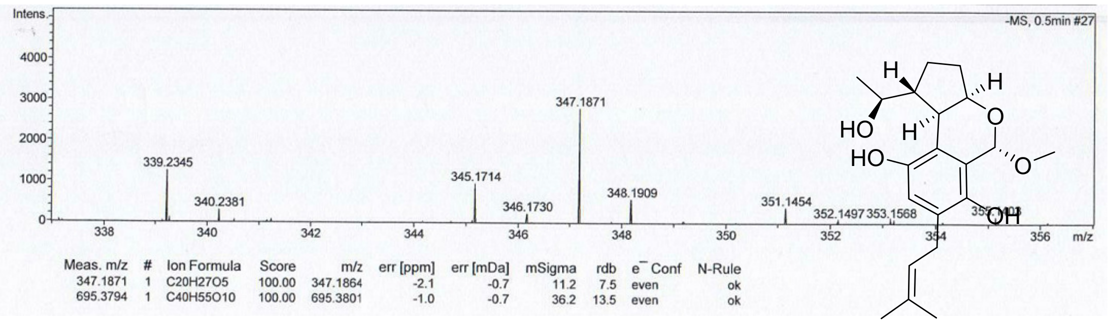

Meas. m/z	#	Ion Formula	Score	m/z	err [ppm]	err [mDa]	mSigma	rdb	e⁻ Conf	N-Rule
347.1871	1	C20H27O5	100.00	347.1864	-2.1	-0.7	11.2	7.5	even	ok
695.3794	1	C40H55O10	100.00	695.3801	-1.0	-0.7	36.2	13.5	even	ok

2

Figure S21 The UV spectrum of euroticin G (**2**).

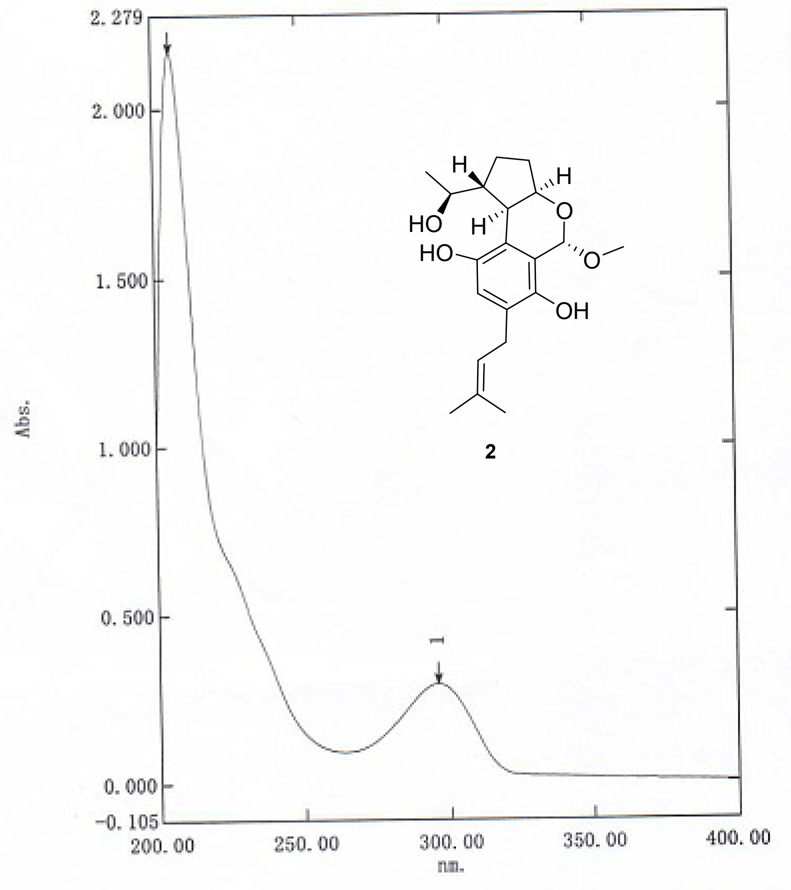

Figure S22 The ^{1}H NMR (500 MHz) spectrum of euroticin H (**3**) in acetone-d_6.

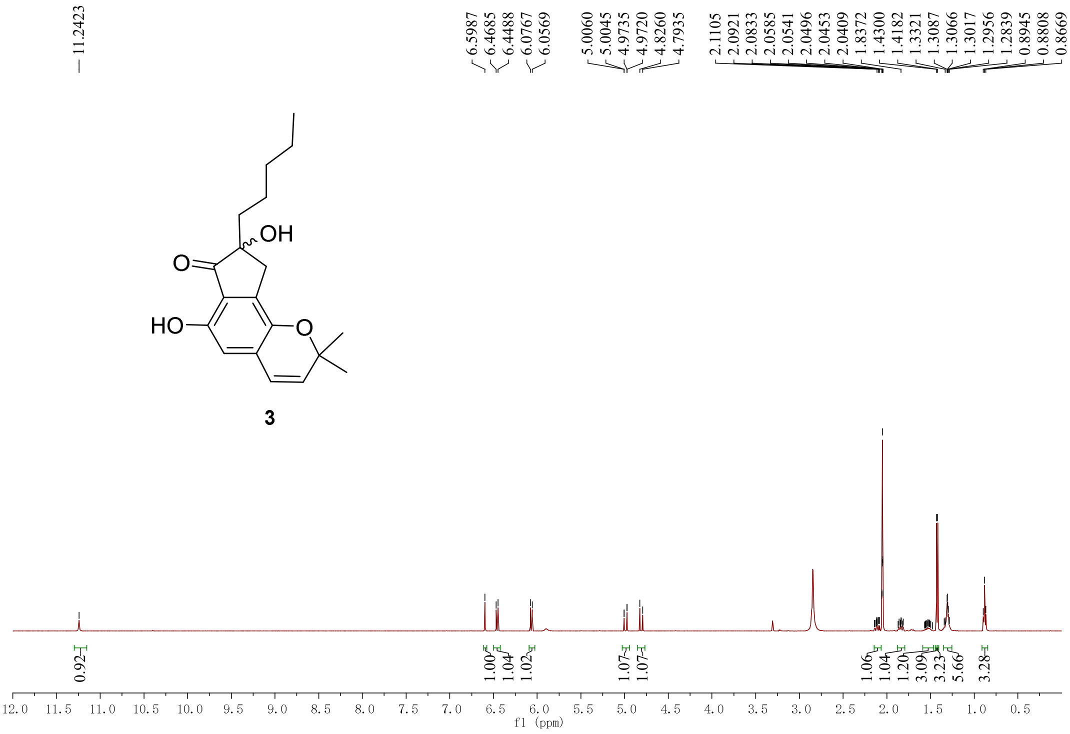

Figure S23 The ^{13}C NMR (125 MHz) spectrum of euroticin H (**3**) in acetone-d_6.

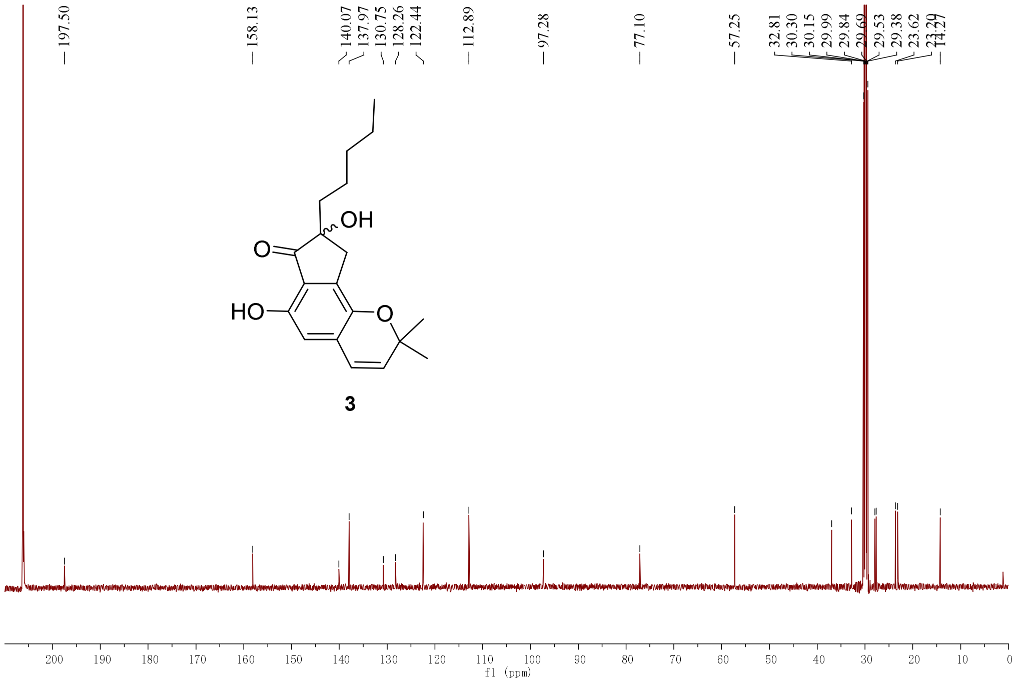

Figure S24 The HSQC (500 MHz) spectrum of euroticin H (**3**) in acetone-*d*6.

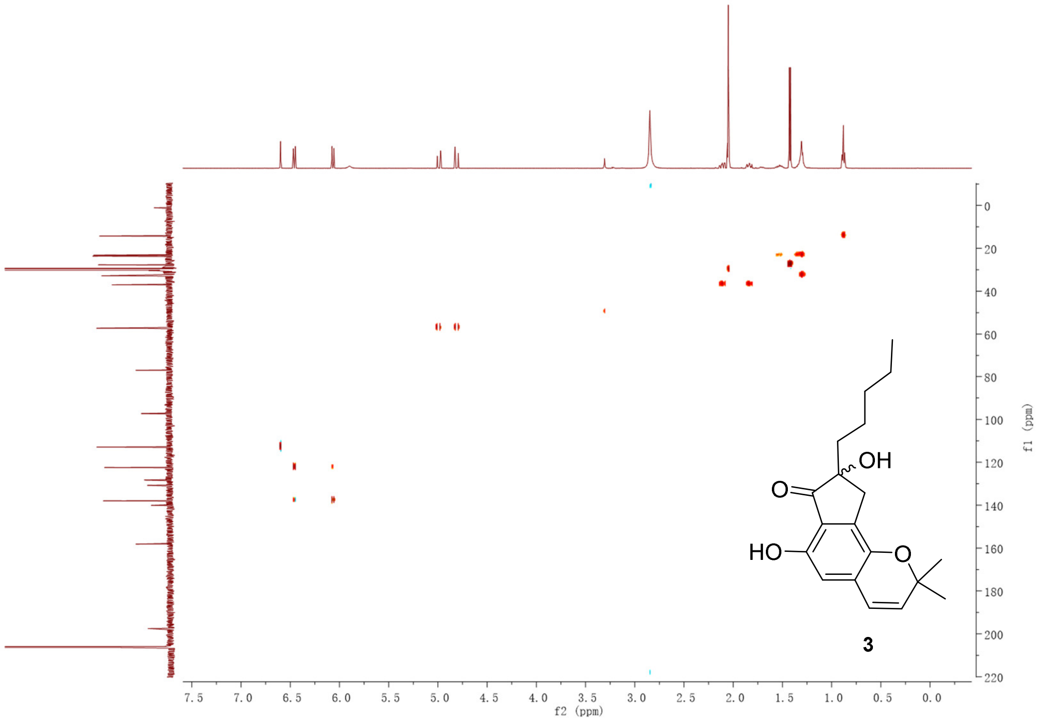

Figure S25 The HMBC (500 MHz) spectrum of euroticin H (**3**) in acetone-*d*6.

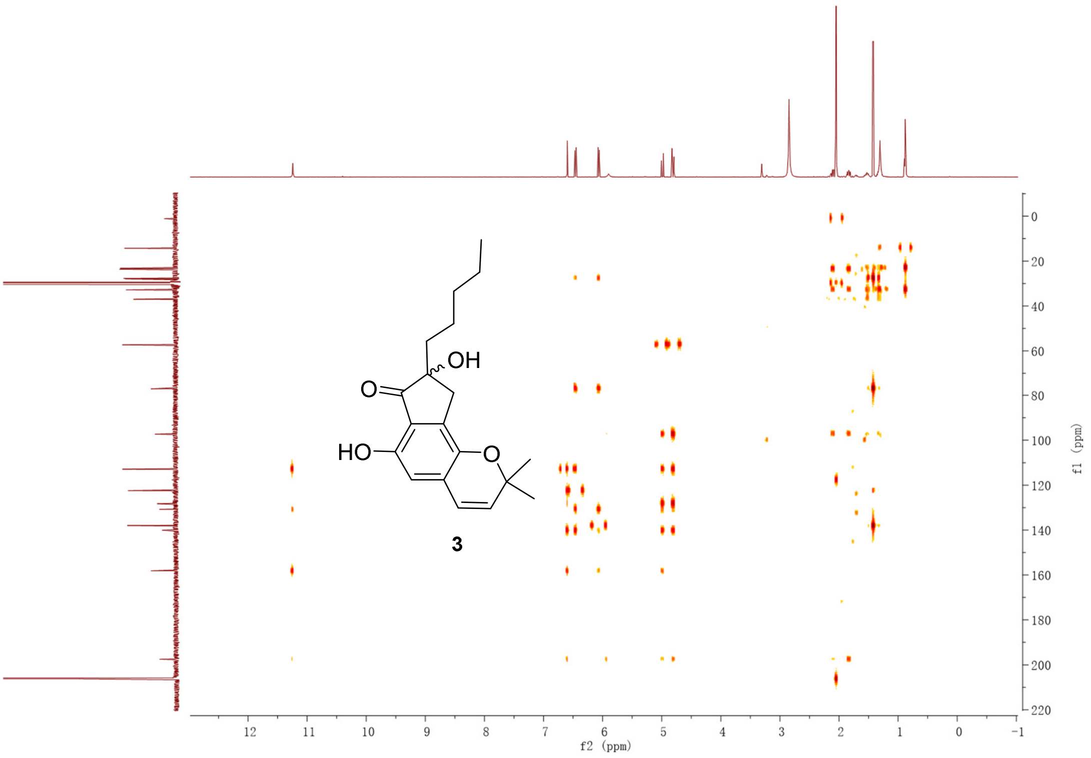

Figure S26 The ^1H-^1H COSY (500 MHz) spectrum of euroticin H (**3**) in acetone-d_6.

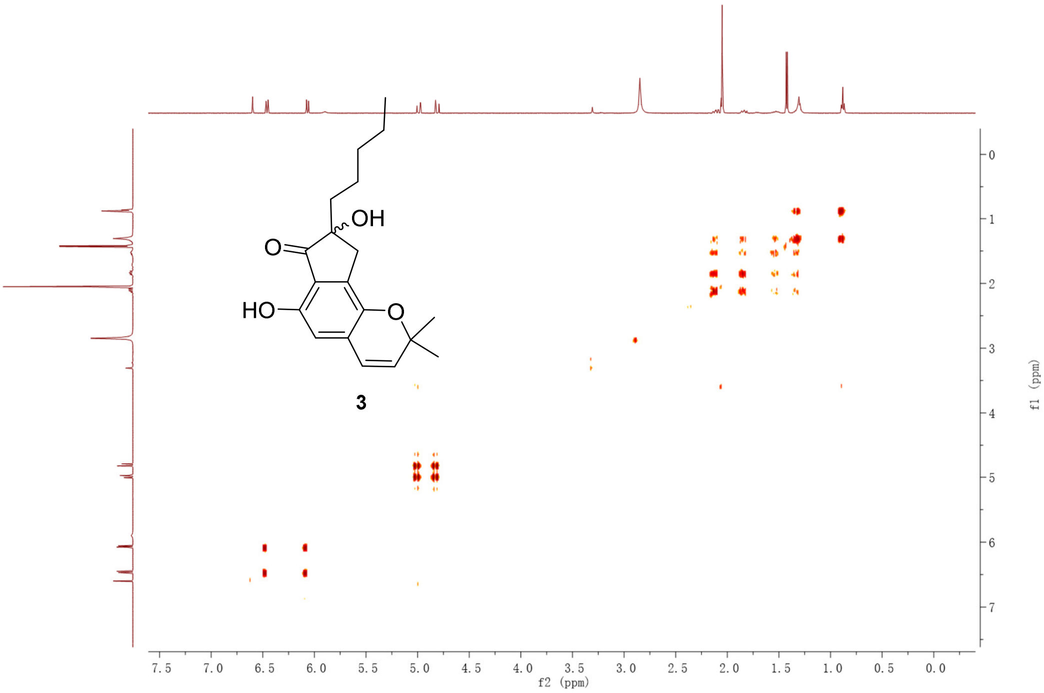

Figure S27 The HRESIMS spectrum of euroticin H (**3**).

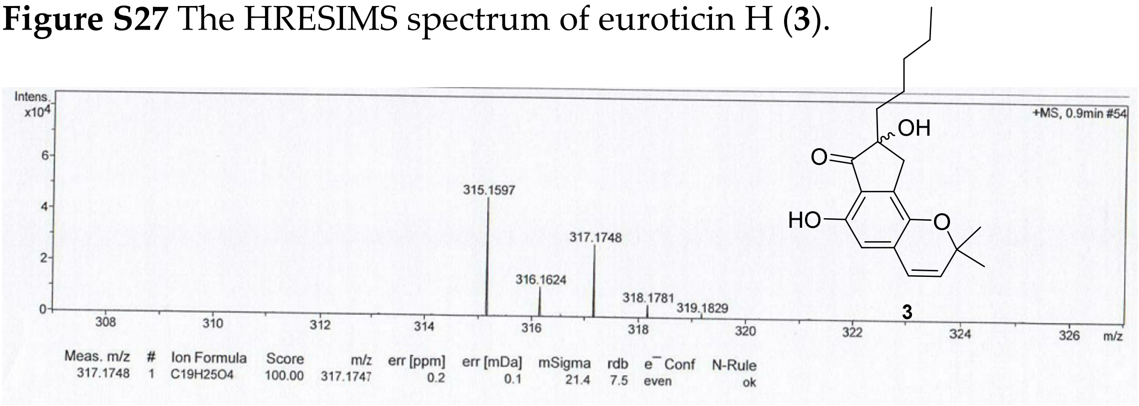

Figure S28 The HRESIMS spectrum of euroticin H (**3**).

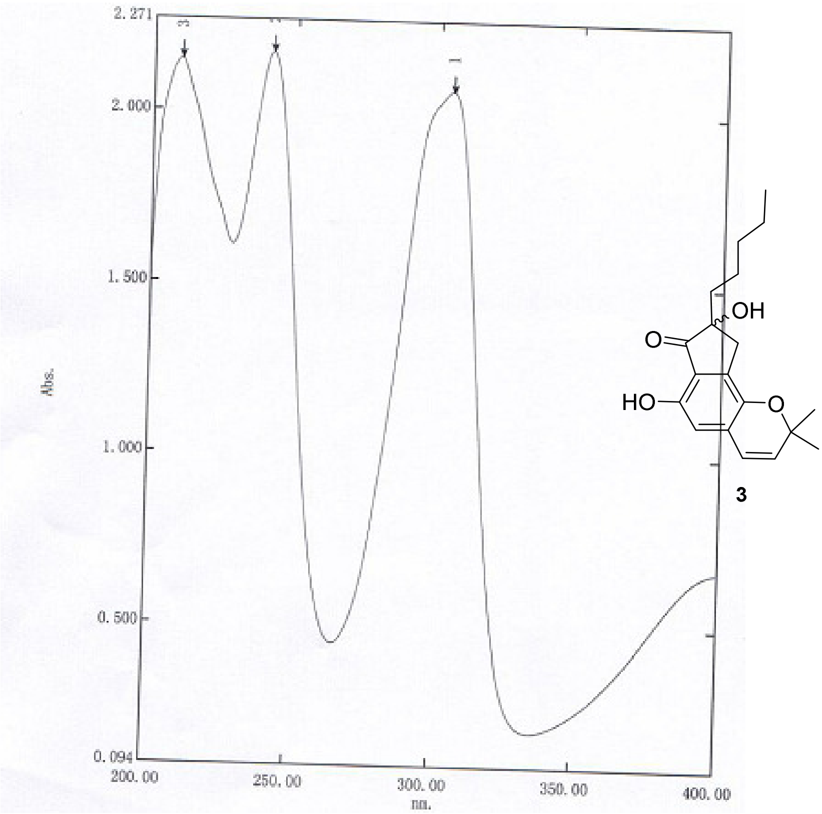

Figure S29 The ^{1}H NMR (700 MHz) spectrum of euroticin I (**4**) in DMSO-d_6.

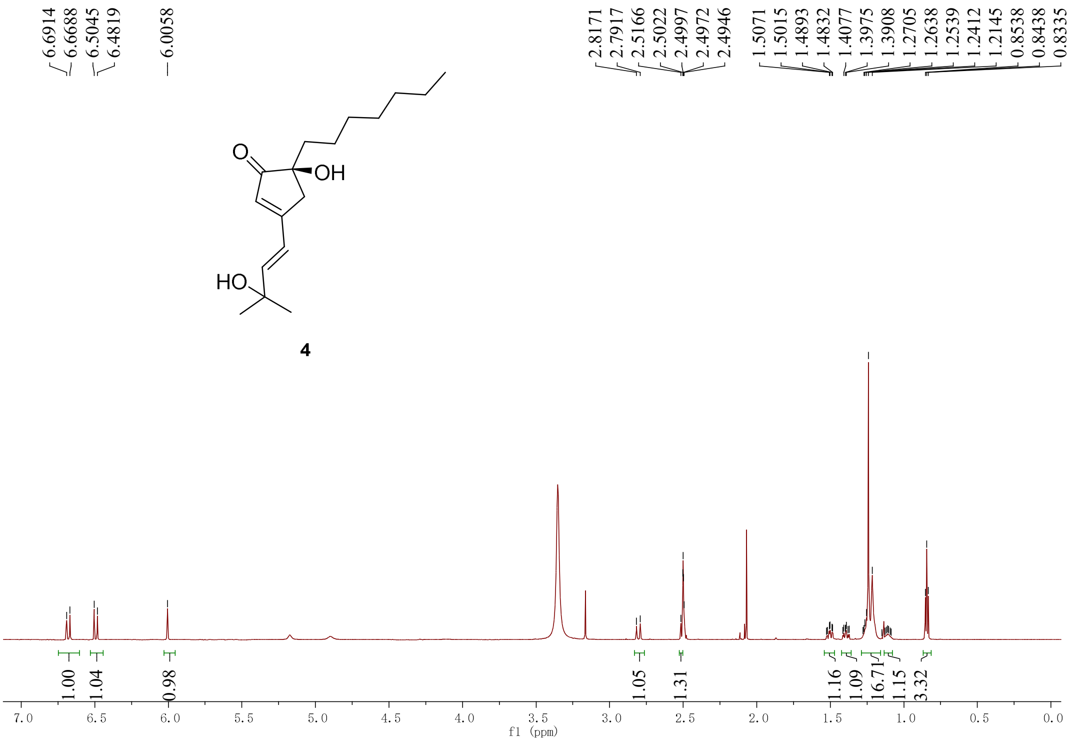

Figure S30 The ^{13}C NMR (175 MHz) spectrum of euroticin I (**4**) in DMSO-*d*$_6$.

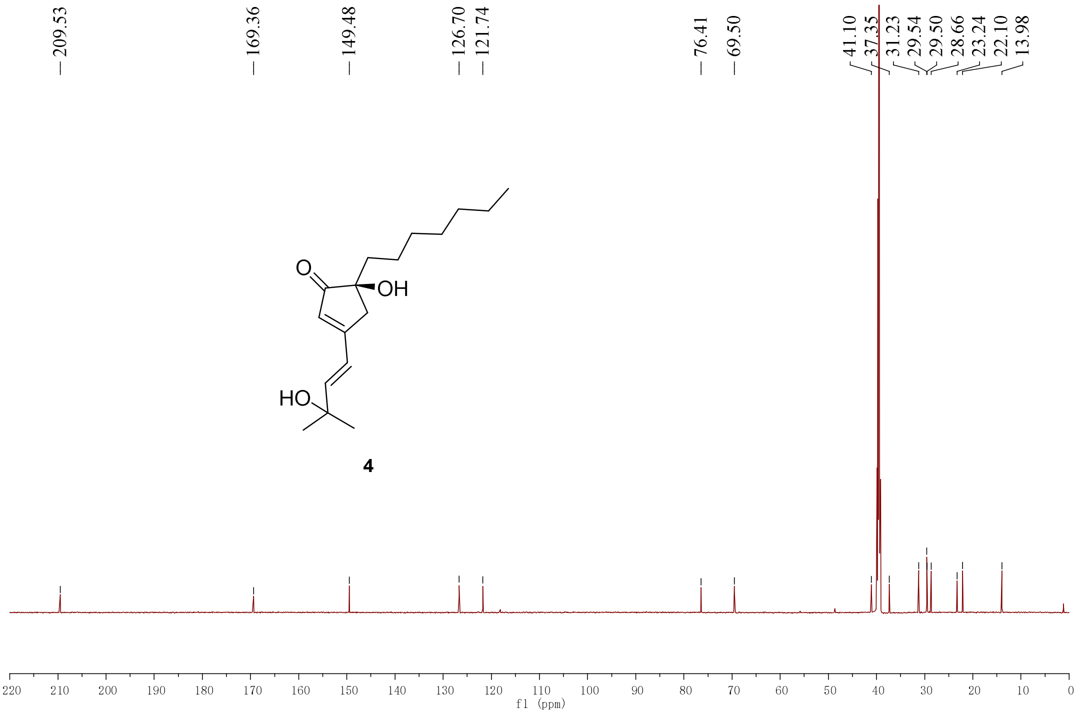

Figure S31 The HSQC (700 MHz) spectrum of euroticin I (**4**) in DMSO-*d*$_6$.

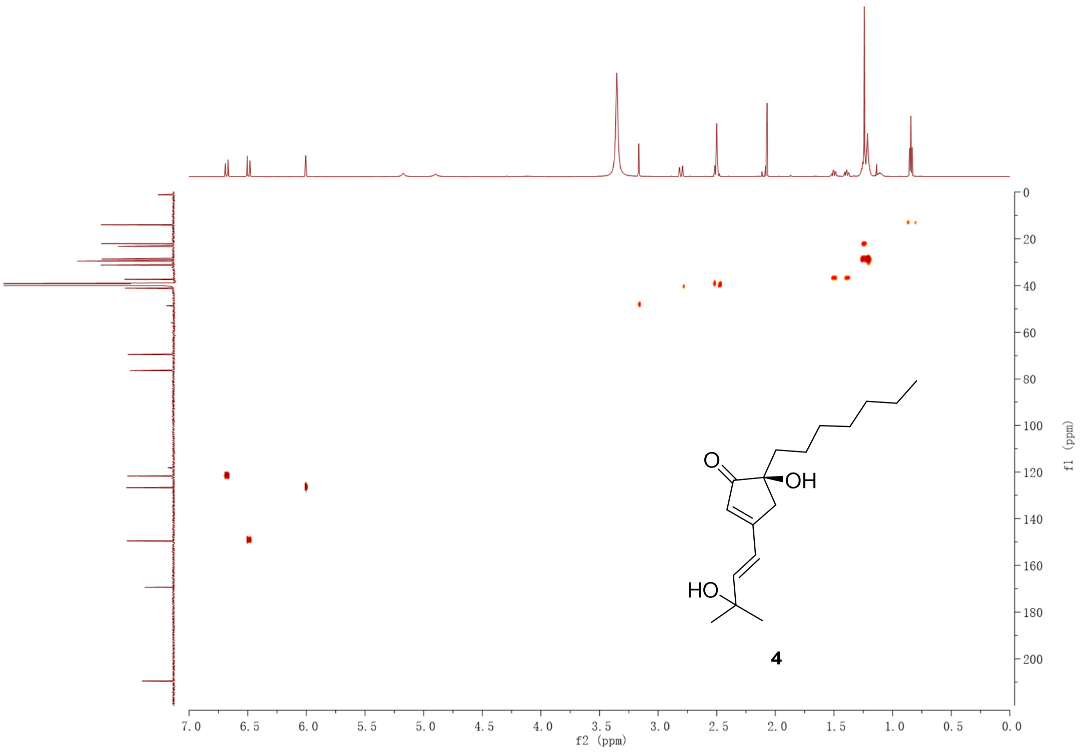

Figure S32 The HMBC (700 MHz) spectrum of euroticin I (**4**) in DMSO-*d*6.

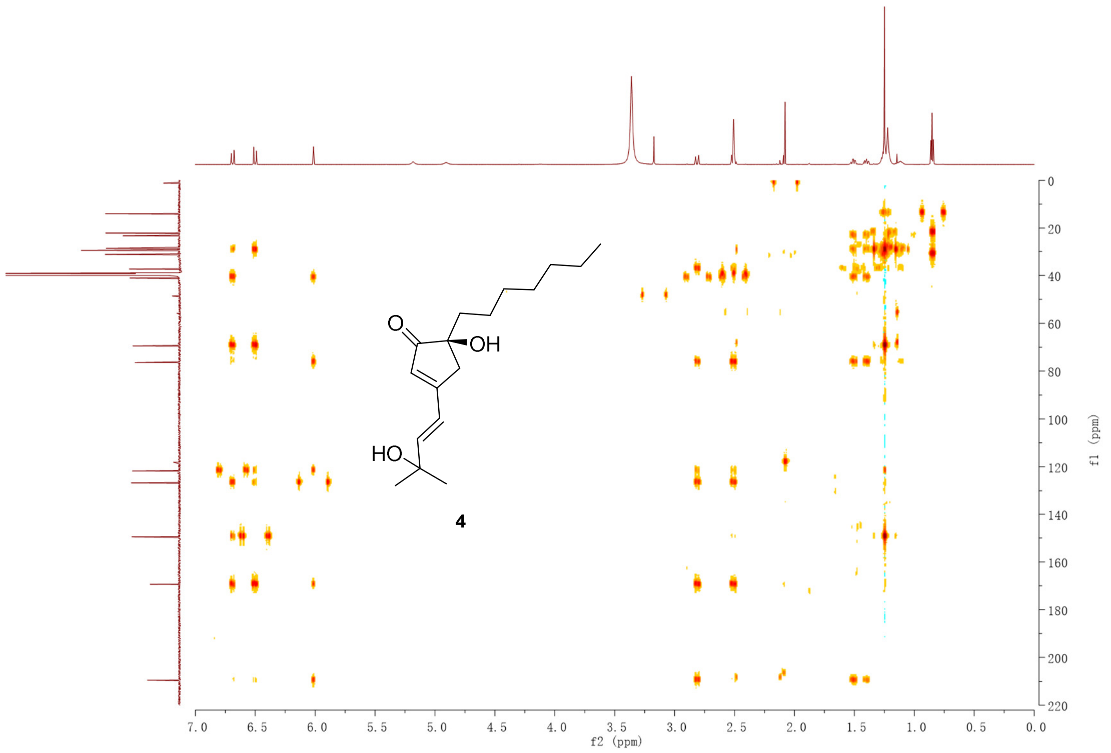

Figure S33 The ¹H-¹H COSY (700 MHz) spectrum of euroticin I (**4**) in DMSO-*d*6.

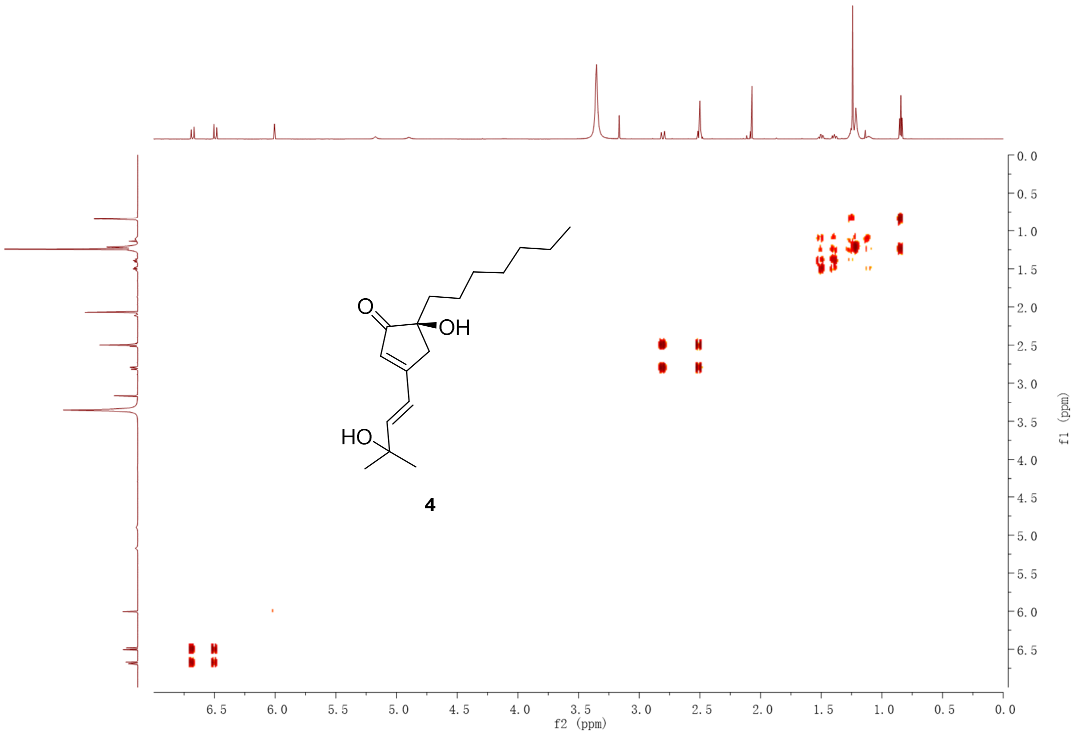

Figure S34 The HRESIMS spectrum of euroticin I (**4**).

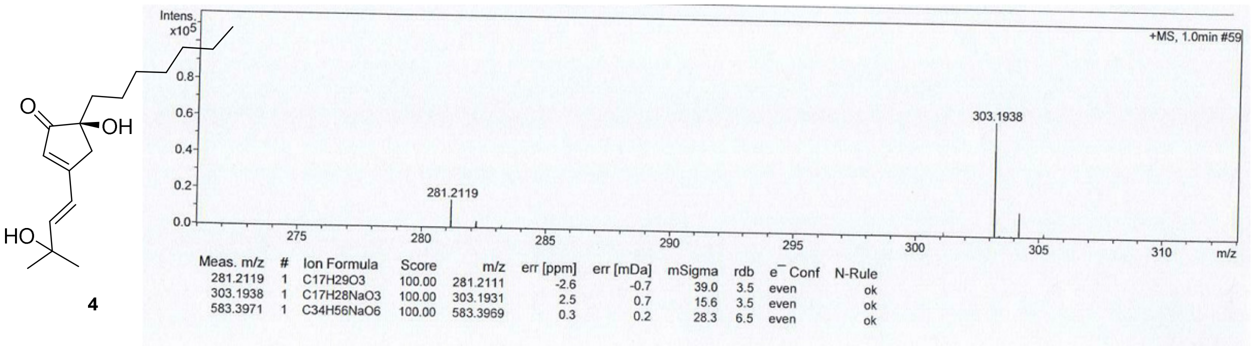

Meas. m/z	#	Ion Formula	Score	m/z	err [ppm]	err [mDa]	mSigma	rdb	e⁻ Conf	N-Rule
281.2119	1	C17H29O3	100.00	281.2111	-2.6	-0.7	39.0	3.5	even	ok
303.1938	1	C17H28NaO3	100.00	303.1931	2.5	0.7	15.6	3.5	even	ok
583.3971	1	C34H56NaO6	100.00	583.3969	0.3	0.2	28.3	6.5	even	ok

Figure S35 The UV spectrum of euroticin I (**4**).

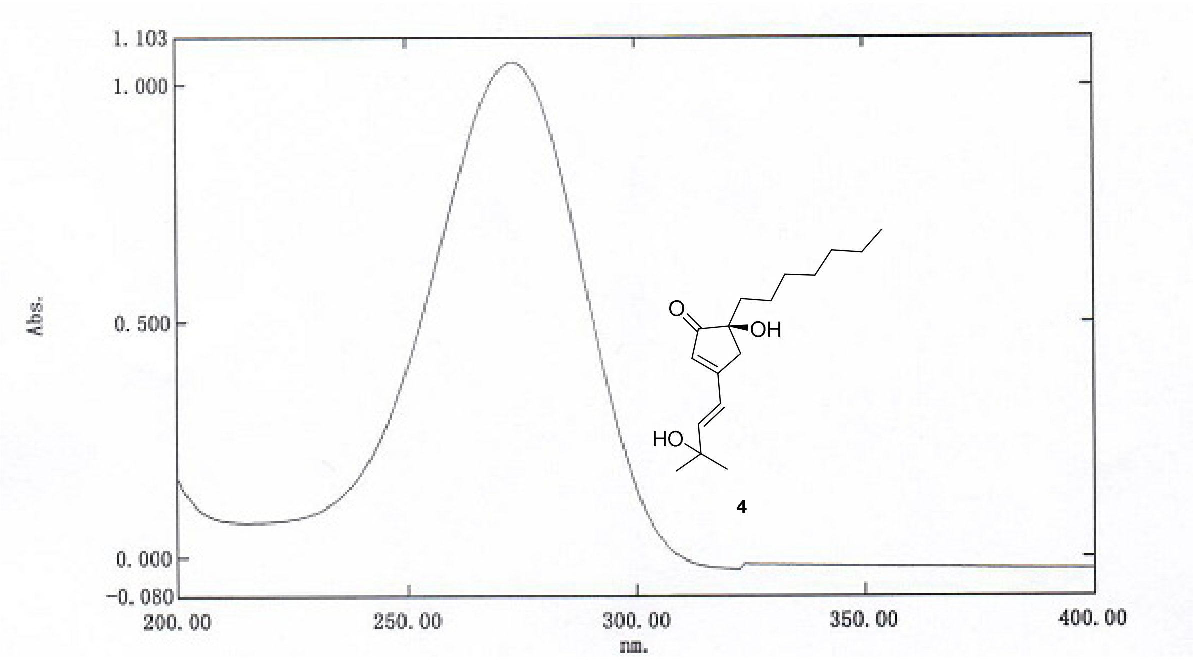

Figure S36 The ^{1}H NMR (700 MHz) spectrum of eurotirumin (**5**) in acetone-d_6.

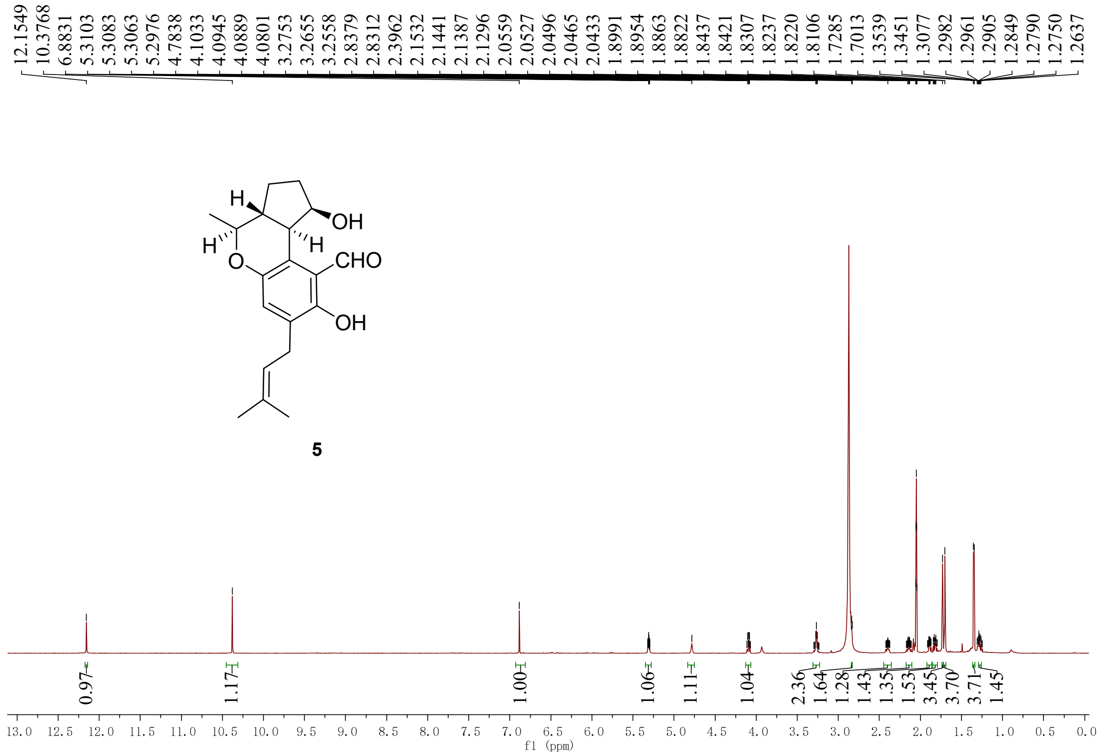

Figure S37 The ^{13}C NMR (175 MHz) spectrum of eurotirumin (**5**) in acetone-d_6.

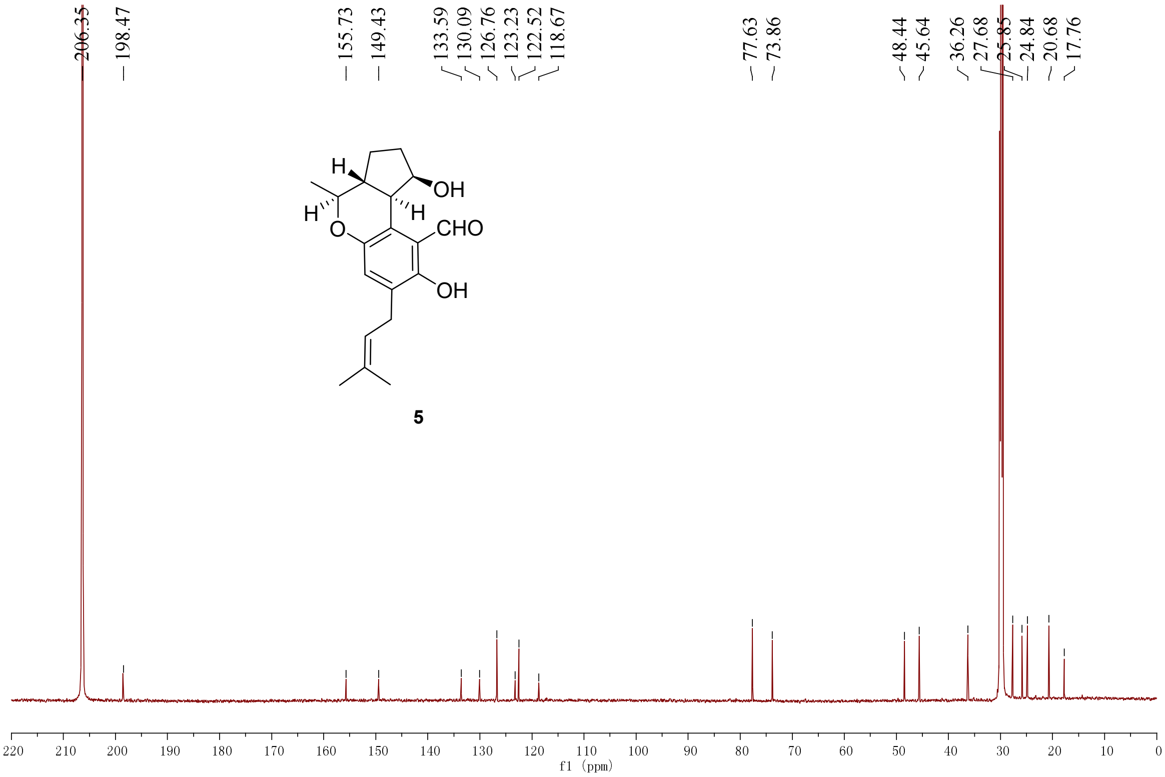

Figure S38 The HSQC (700 MHz) spectrum of eurotirumin (**5**) in acetone-*d*6.

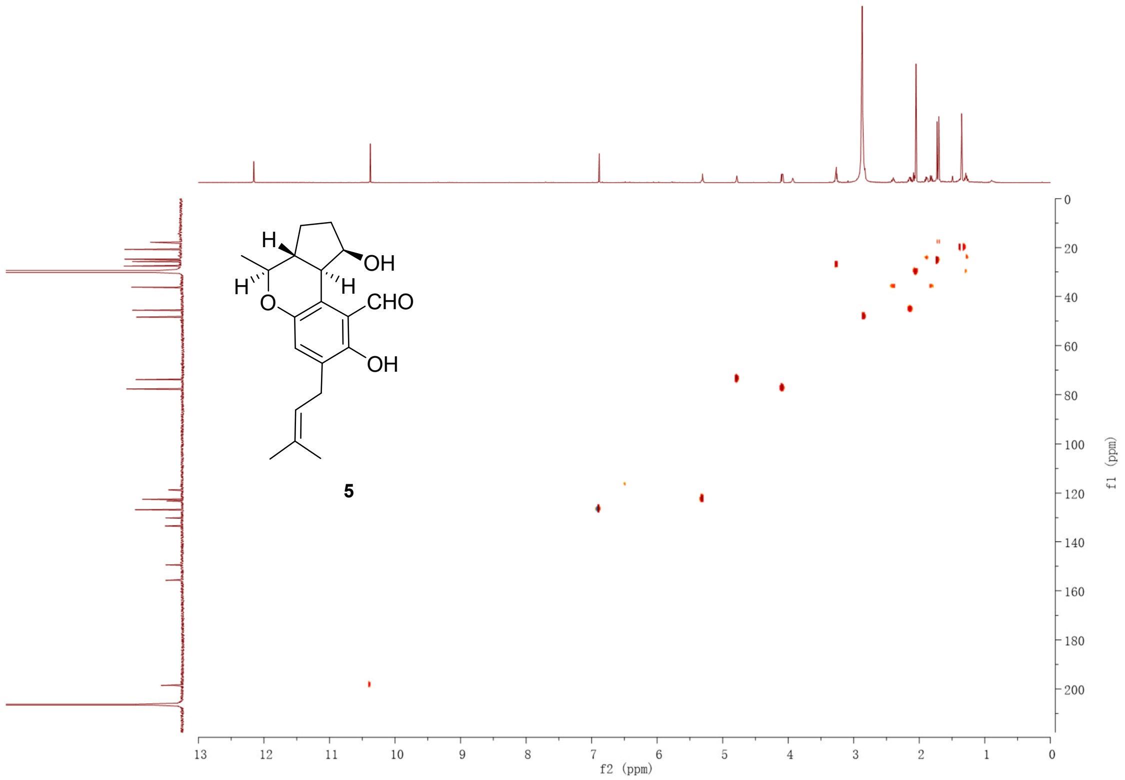

Figure S39 The HMBC (700 MHz) spectrum of eurotirumin (**5**) in acetone-*d*6.

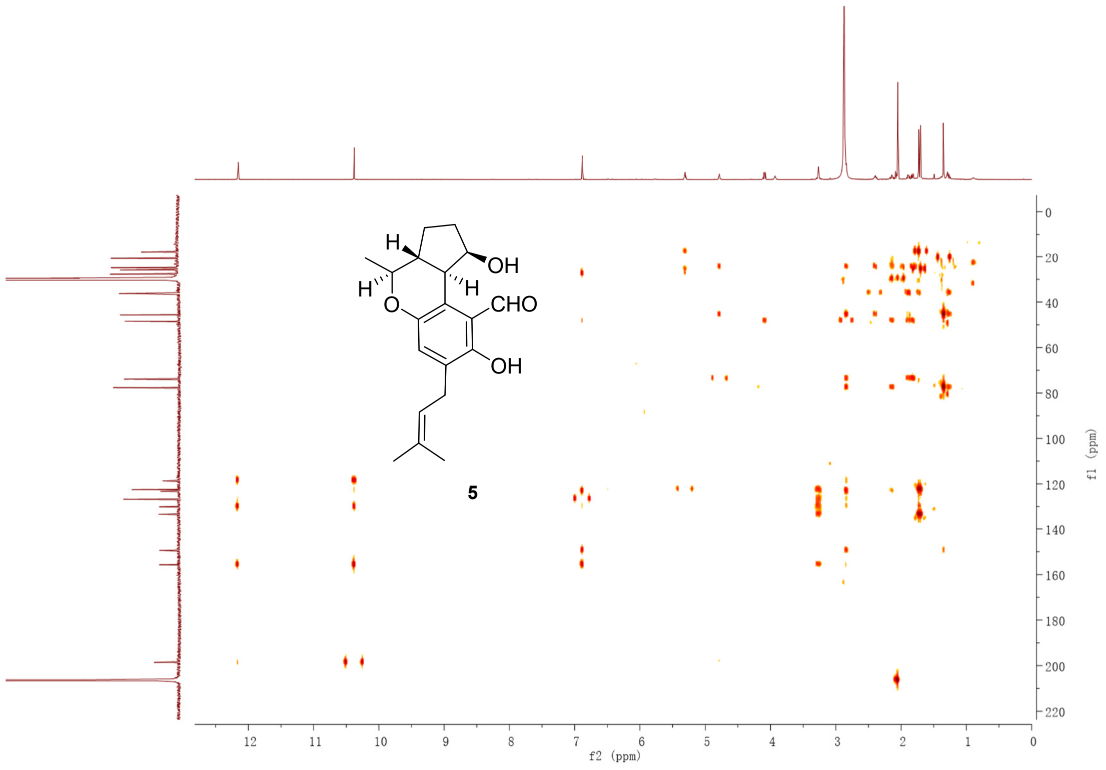

Figure S40 The ^1H-^1H COSY (700 MHz) spectrum of eurotirumin (**5**) in acetone-d_6.

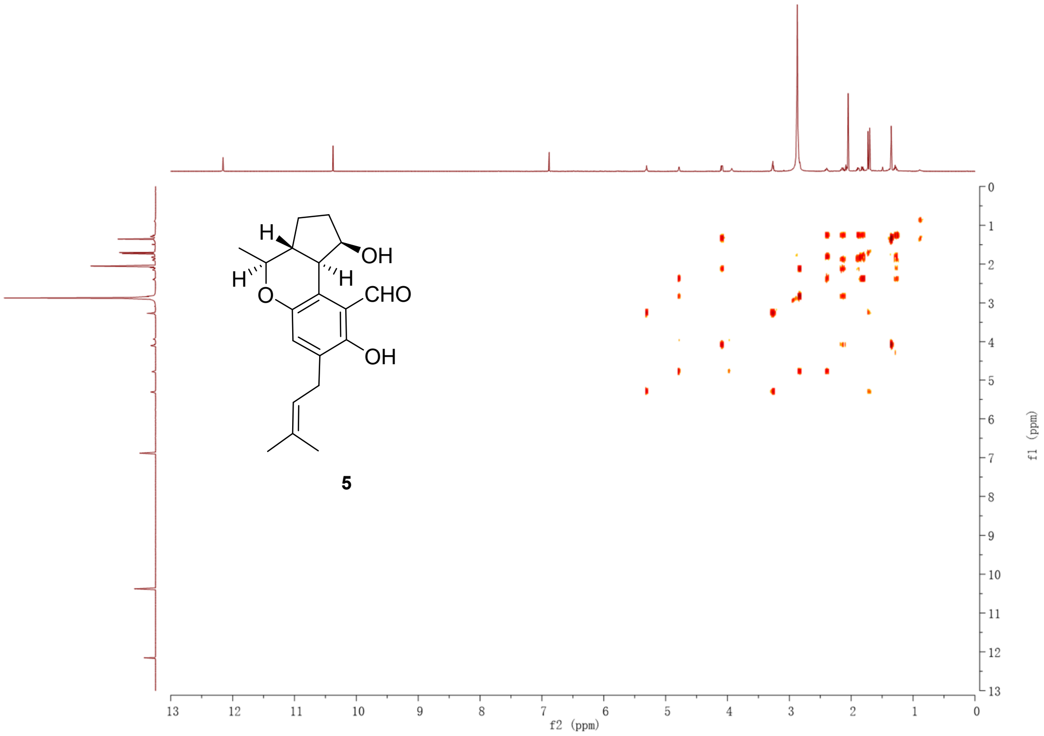

Figure S41 The ROESY (700 MHz) spectrum of eurotirumin (**5**) in acetone-d_6.

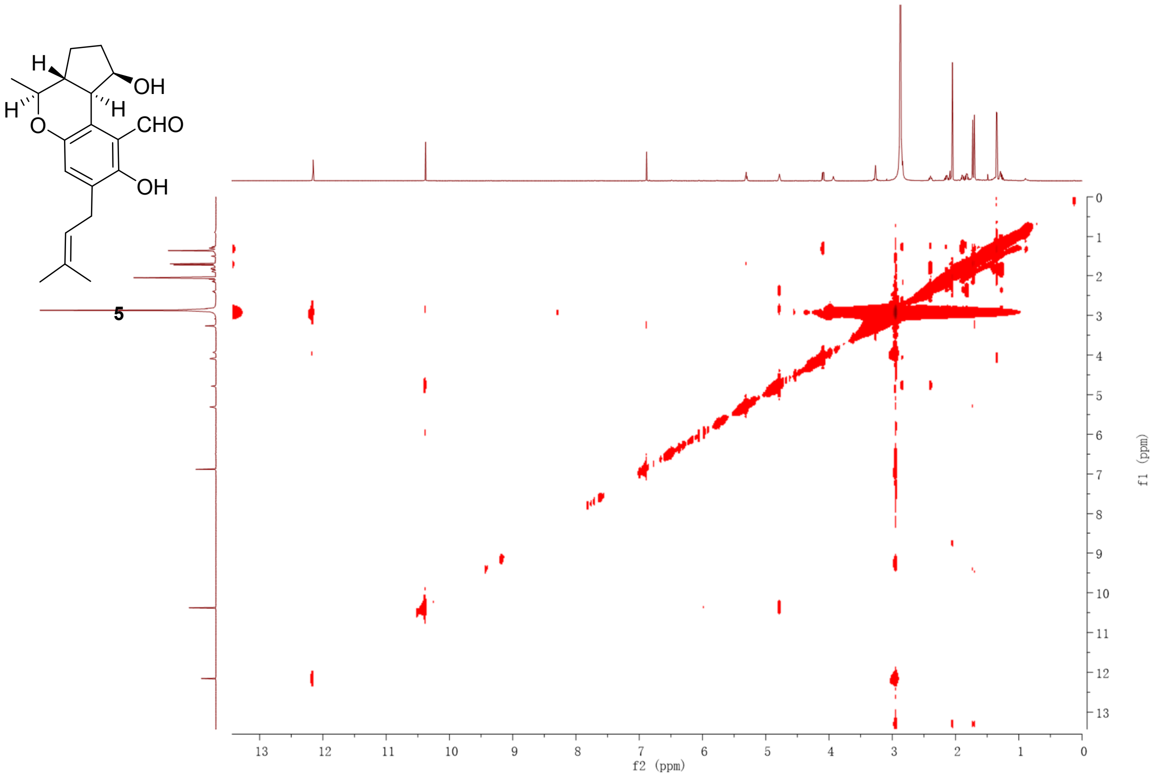

Figure S42 The HRESIMS spectrum of eurotirumin (**5**).

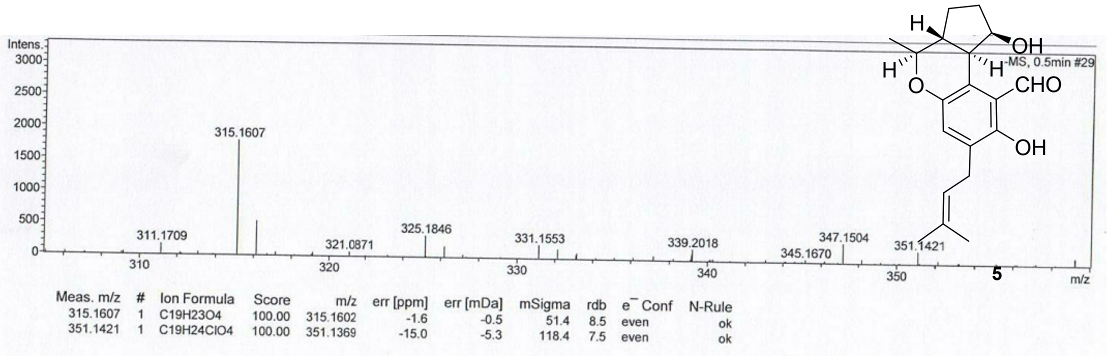

Figure S43 The UV spectrum of eurotirumin (**5**).

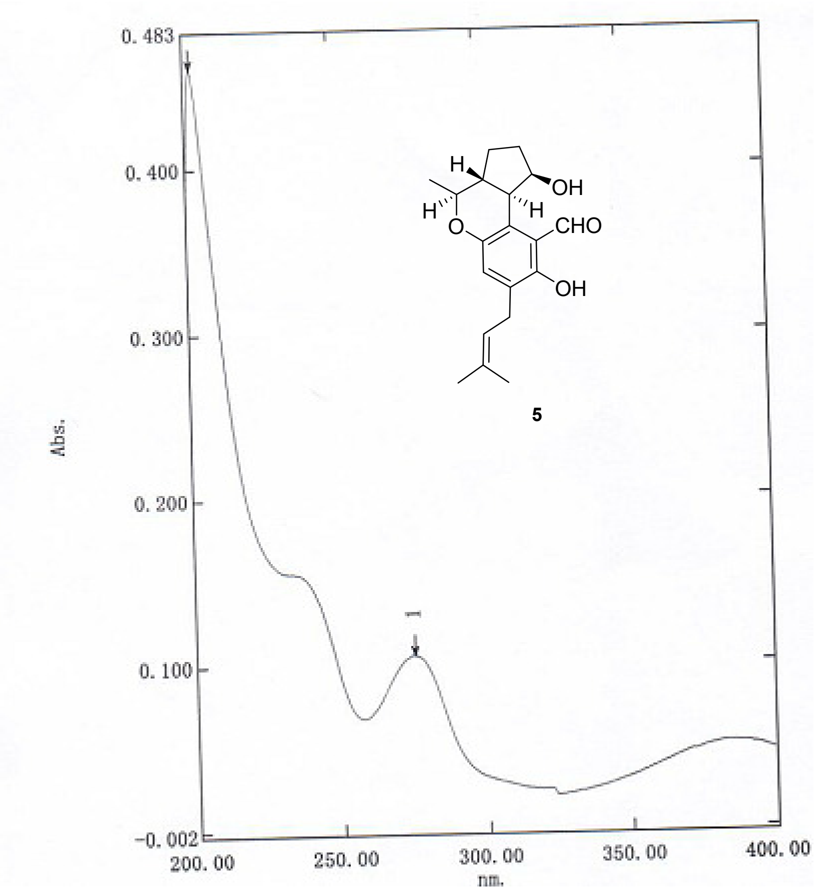

Supplement: Supplementary file 1 [file marinedrugs-19-00543-s001.zip › marinedrugs-1379758-supplementary.pdf]
